# Separate transcription and splicing gene networks are linked and coordinated by the pRb–E2F pathway

**DOI:** 10.1093/nar/gkag016

**Published:** 2026-01-30

**Authors:** Simon M Carr, Geng Liu, Wojciech Barczak, Hasan Syffudin, Shonagh Munro, Denise Reyna-Jeldes, Iolanda Vendrell, Benedikt Kessler, Adam P Cribbs, Alexander Kanapin, Anastasia Samsonova, Nicholas B La Thangue

**Affiliations:** Laboratory of Cancer Biology, Department of Oncology, University of Oxford Old Road Campus Research Building, Oxford OX3 7DQ, United Kingdom; Laboratory of Cancer Biology, Department of Oncology, University of Oxford Old Road Campus Research Building, Oxford OX3 7DQ, United Kingdom; Laboratory of Cancer Biology, Department of Oncology, University of Oxford Old Road Campus Research Building, Oxford OX3 7DQ, United Kingdom; Laboratory of Cancer Biology, Department of Oncology, University of Oxford Old Road Campus Research Building, Oxford OX3 7DQ, United Kingdom; Laboratory of Cancer Biology, Department of Oncology, University of Oxford Old Road Campus Research Building, Oxford OX3 7DQ, United Kingdom; IngenOx Therapeutics Ltd, Magdalen Centre, Oxford Science Park, Oxford OX4 4GA, United Kingdom; Laboratory of Cancer Biology, Department of Oncology, University of Oxford Old Road Campus Research Building, Oxford OX3 7DQ, United Kingdom; Target Discovery Institute, Centre for Medicines Discovery, Nuffield Department of Medicine, University of Oxford, Oxford OX3 7FZ, United Kingdom; Target Discovery Institute, Centre for Medicines Discovery, Nuffield Department of Medicine, University of Oxford, Oxford OX3 7FZ, United Kingdom; Nuffield Department of Orthopaedics, Rheumatology and Musculoskeletal Sciences, University of Oxford, Oxford OX3 7LD, United Kingdom; Oxford Translational Myeloma Centre (OTMC), Nuffield Department of Orthopaedics, Rheumatology and Musculoskeletal Sciences, University of Oxford, Oxford OX3 7LD, United Kingdom; Department of Oncology, University of Oxford, Old Road Campus Research Building, Oxford OX3 7DQ, United Kingdom; Department of Oncology, University of Oxford, Old Road Campus Research Building, Oxford OX3 7DQ, United Kingdom; Laboratory of Cancer Biology, Department of Oncology, University of Oxford Old Road Campus Research Building, Oxford OX3 7DQ, United Kingdom

## Abstract

The pRb–E2F pathway is involved in mediating diverse cell fates, and oncogenic disruption of the pathway is regarded as a hallmark of cancer. Recent studies highlighted the pRb–E2F axis as a regulator of a large gene network which includes RNA splicing and transcription targets. Here, we have performed a deep genome-wide analysis of differentially expressed genes (DEGs) and alternatively spliced (AS) RNA targets which highlighted broadly non-overlapping networks of genes that are independently regulated by the pRb–E2F pathway. Individual pathway components, including E2F1, pRb, and PRMT5, either as single or combined knockouts, were found to influence DEG and AS networks but to different extents. An analysis of the E2F1 interactome revealed SRSF2 and HNRNPC as candidate proteins that were able to functionally assist E2F1 in mediating AS events. Moreover, E2F1 AS activity was evident as cells progress through the cell cycle and during the DNA damage response, and apparent in tumour models. Our results highlight gene networks where transcription and splicing are linked and coordinated by the pRb–E2F pathway, and further establish the widespread influence that pRb, E2F1, and PRMT5 have on regulating biological diversity through RNA splicing control.

## Graphical Abstract

**Graphical Abstract:**
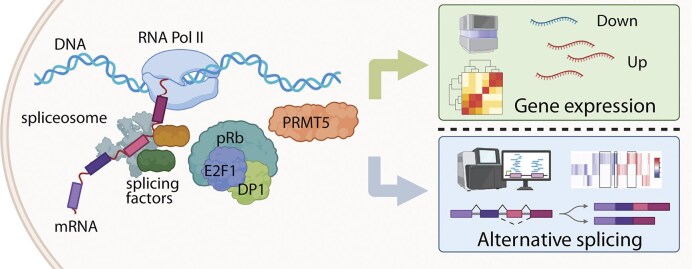

## Introduction

The ability to coordinate cell growth and division with gene expression is an intrinsic and fundamental property of metazoan cells. E2F is a generic term for a family of widely expressed and evolutionarily conserved transcription factors that are master regulators responsible for integrating growth and division with the transcription apparatus during cell cycle progression and cell cycle exit [1–5]. E2F is a key physical target for the retinoblastoma tumour suppressor protein (pRb) which, in normal cells, allows pRb to effect transcriptional control of target genes and thereby influence cell cycle progression [6–9]. Because of the frequent mutation of the RB1 gene or aberrant control by its upstream cyclin/CDK regulators, the pRb–E2F pathway is regarded to be of utmost importance to human cancer [6, 8, 10]. In fact, deregulation of the pathway is widely regarded to be one of the hallmarks of human cancer [11, 12].

In normal cells, most generic E2F activity exists as a complex mixture of heterodimeric transcription factors, where an E2F subunit is bound to a DP subunit [1–4]. There are eight related E2F subunit genes in the human genome that are grouped according to functional and sequence similarity, with roles ascribed to cell cycle control and diverse types of cell fate [1–4]. E2F1 was the first subunit to be identified and is consequently the best characterized, with DP1 the most frequent heterodimeric DP family member seen in mammalian cells in complex with E2F1 [13–16]. Classical E2F target genes fall into different functional categories, such as the well-characterized cell cycle, DNA synthesis, and apoptotic targets, but include other groups involved with metabolic control, differentiation, senescence, and autophagy [1–5, 17–21].

The E2F1 subunit is a target for diverse types of post-translational control [9, 20]. For example, the protein arginine methyl-transferase (PRMT) 5, which is often under abnormal control in cancer [22, 23], methylates E2F1 switching E2F1 from its focused role in transcriptional control to one that has an expanded repertoire on diverse transcription targets [24–29]. For example, pharmacological inhibition of PRMT5 and manipulation of E2F1 activity alter the genes expressed from the non-coding genome [24]. Furthermore, PRMT5 methylation enables E2F1 to regulate a diverse group of genes at the level of alternative RNA splicing (AS), as opposed to its classical transcription-based mechanisms [28]. This is likely to reflect the role of arginine methylation in mediating interactions between E2F1 and components of the splicing machinery, for example SND1/TSN, which ‘reads’ the methylation mark on E2F1 allowing an influence on the splicing process [28, 29]. It is established that AS can regulate many biological processes and plays an important role in diseases including cancer [30, 32, 33]. The ability to impact on AS thus provides the pRb–E2F pathway with an even greater influence on cellular processes in addition to that already provided through transcriptional control.

RNA splicing itself is a highly regulated process, involving the activity of the core spliceosome and additional splicing factor (SF) proteins. Whilst the spliceosome recognizes core sequences in the pre-mRNA that mark exon–intron boundaries, another layer of regulation is provided by SF proteins, which recognize *cis-*acting enhancer or silencer sequences in exons and introns [30–33]. Binding of SFs to these sequences either enhance or inhibit the spliceosome from including an exon into mature mRNA [30–33]. The serine/arginine-rich (SR) proteins and the heterogeneous nuclear ribonucleoproteins (hnRNPs) represent two SF protein families that enhance or repress exon inclusion [30–33]. SR proteins are generally regarded as positive regulators of splicing, whilst hnRNPs generally repress exon inclusion [30–33], though in reality both families of SFs can either promote or repress splicing when binding different sequences in pre-mRNA [30–33].

Here, we have explored the role of the pRb–E2F pathway focussing on its key protein components and measured the influence on alternative splicing and transcription. By performing extensive genome-wide expression analysis in pRb and E2F1 KO cells together with pharmacological inhibition of PRMT5, we found that each component impacted on transcription and AS of target gene networks. However, we observed significant differences in the extent and type of genes affected by each pathway component and, importantly, we found that transcription and AS targets fell into distinct groups of genes. Further, we identified certain AS events that are under cell cycle control, where the integrity of the pRb–E2F pathway was necessary for the timely appearance of the splicing event during the cell cycle. The splicing and RNA processing factors, SRSF2 and HNRNPC, were shown to be necessary for E2F to influence AS events. Our results establish that control of AS is a general property of the pRb–E2F pathway that has a wide influence on diverse gene sets. Significantly, AS operates hand in hand with transcription and thus enables E2F regulated gene expression to be further fine-tuned through AS to cellular requirements.

## Materials and methods

### Cell line generation, culture, and compound treatments

Generation of human p53-/- HCT116 E2F1 CRISPR and CAS9 control cells has been described previously [25]. MCF7 and PANC1 CRISPR cell lines were generated using the method described by Ran *et al.* [34]. Genomic DNA was extracted (QIAamp DNA Mini Kit, Qiagen, Hilden, Germany), and a small sequence surrounding the Cas9 cleavage site in *E2F1* or *RB1* was PCR amplified prior to sequencing to confirm CRISPR knockout of the gene. Mouse Colon26 cells were used by Charles Rivers Laboratories (Wilmington, USA) for out-sourced tumour challenge models [24]. HCT116, PANC1, and MCF7 cells were cultured in Dulbecco’s Modified Eagle Medium (DMEM) (Sigma–Aldrich, St. Louis, USA), supplemented with 10% foetal bovine serum (Labtech, London, UK) and 1% penicillin/streptomycin (Gibco, Waltham, USA). All cell lines were tested for mycoplasma contamination before use.

Selective PRMT5 inhibitor (T1-44) (synthesized by Argonaut Therapeutics, Oxford, UK) has been described and characterized previously [24, 25]. It demonstrated potent inhibition of PRMT5 in *in vitro* enzyme assays, with minimal activity against other PRMTs or lysine methyltransferases [24]. It was used for 48 h at 1 μM final concentration unless otherwise stated. For comparison, established inhibitors of PRMT5, JNJ-64619178, and LLY-283, were used (Selleck, Houston, USA). For splicing analysis under different cell cycle conditions, cells were either treated with 20 μM etoposide (Stratech Scientific, Ely, UK) for 24–48 h or with 1 mM hydroxyurea (Fisher Scientific, Waltham, USA) for 24 h, prior to PBS washout and replacement with fresh media for 8 h. Cell cycle synchronization was performed using a double thymidine block [2 mM thymidine (Fisher Scientific, Waltham, USA) for 18 h, followed by a PBS washout and release for 10 h, followed by a second thymidine block for 15 h, then a subsequent PBS washout and release for up to 12 h].

### siRNA transfections

RNA interference was performed with 20 nM siRNA for 72 h using the Oligofectamine transfection reagent (Invitrogen, Waltham, USA), as per the manufacturer’s instructions. SRSF2 (s12730) and HNRNPC (s6719) siRNAs were purchased from Life Technologies. The non-targeting control siRNA sequence was 5′-AGCUGACCCUGAAGUUCUU-3′ (Life Technologies, Carlsbad, USA).

### Immunoblots and antibodies

For immunoblots, cells were harvested in modified RIPA buffer [50 mM Tris–HCl pH 7.5, 150 mM NaCl, 1% Igepal CA-630 (v/v), 1 mM EDTA, 1 mM NaF, 1 mM Na_3_VO_4_, 1 mM AEBSF, and protease inhibitor cocktail] and incubated on ice for 30 min prior to SDS–PAGE and transfer to nitrocellulose. The following antibodies were used in immunoblots: β-actin (clone AC-74, Sigma–Aldrich, St. Louis, USA), E2F1 (3742S, Cell Signaling Technology, Danvers, USA), GAPDH (clone 6C5, MAB374, Millipore, Burlington, USA), HNRNPC1/C2 (clone 4F4, sc-32308, Santa Cruz, Dallas, USA), SC35 (EPR12238, ab204916, Abcam, Cambridge, UK), SRSF2 (PA5-12402, Thermo Fisher, Waltham, USA), pRb (clone 4H1, 9309S, Cell Signaling Technology, Danvers, USA), DP-1 (clone TFD-10, sc-53642, Santa Cruz, Dallas, USA), and symmetric di-methyl arginine (SDMe) (13222S, Cell Signaling Technology, Danvers, USA). For all SDMe immunoblots in the manuscript, the image displays the symmetric methylated protein band corresponding to a size of 16 kDa, representing methylated SmD (SNRNPD).

### RNA isolation and quantitative RT-PCR

RNA was isolated from cells using TRIzol (Thermo Fisher, Waltham, USA) according to the manufacturer’s instructions. One microgram of total RNA was used for complementary DNA (cDNA) synthesis. Reverse transcription with oligo (dT)20 (Invitrogen, Waltham, USA) was performed using SuperScript III Reverse Transcriptase (Invitrogen, Waltham, USA) as per the manufacturer’s instructions. Quantitative PCR (qPCR) was then carried out in technical triplicate using the indicated primer pairs (Supplementary Table S1) and the Brilliant III SYBR Green qPCR Master Mix (Stratagene, San Diego, USA) on an AriaMx (Agilent, Santa Clara, USA) instrument. Results were expressed as average (mean) relative mRNA expression as compared to glyceraldehyde-phosphate dehydrogenase (GAPDH) internal calibrator levels using the Δ*C*_t_ method from at least three biological repeat samples. Error bars represent SD unless otherwise indicated. For splicing analysis, forward primers were designed across exon–exon junctions so that they specifically measured the exclusion (i.e. exon 1–exon 3 spanning primer) or the inclusion (i.e. exon 2–exon 3 spanning primer) of a specific exon of interest (in this case exon 2). These primers were paired with a common reverse primer (i.e. contained within exon 3) in the QPCR reaction. The signal from these primer sets was first normalized to GAPDH using the formula: Δ*C*_t_ = GAPDH *C*_t_ – inclusion or exclusion *C*_t_. The inclusion/exclusion ratio was then calculated with the subsequent formula: ratio = 2^[Δ*C*_t_ inclusion – Δ*C*_t_ exclusion], as described previously [35]. Results were expressed as average (mean) values from at least three biological repeat samples.

### Immunoprecipitation

HCT116 WT E2F1 and HCT116 E2F1 Cr cells were seeded into dishes and treated with 1 μM PRMT5 inhibitor (T1-44) for 48 h as appropriate. Cells were then harvested in modified RIPA buffer and incubated on ice for 30 min. Extracts were then pre-cleared by mixing with 50 μl of TrueBlot Ig IP agarose beads (Rockland Immunochemicals, Pottstown, USA) and 3 μg of non-specific IgG for 3–4 h at 4°C. Cleared extracts were then divided equally into tubes and mixed with 3 μg of E2F1 specific IgG (KH-95, sc-251, Santa Cruz, Dallas, USA; or clone G10, Argonaut Therapeutics, Oxford, UK) overnight. 50 μl of TrueBlot Ig IP agarose beads were added for a further 2 h, prior to washing the beads four times in modified RIPA buffer. Immunoprecipitates were eluted using 100 μl of SDS–PAGE loading buffer and boiling for 5 min.

### Chromatin immunoprecipitation (ChIP)

E2F1 chromatin immunoprecipitation (ChIPs) were performed as described previously [24], using 3 μg of appropriate antibody [control rabbit IgG, anti-E2F1 (A300-766A), Bethyl Laboratories, Montgomery, USA] and pre-blocked protein A/G beads. The recovered DNA was purified and QPCR was performed in technical triplicate with Brilliant III Ultra-Fast SYBR green QPCR master mix on an AriaMx QPCR instrument (Agilent, Santa Clara, USA) using primers flanking predicted E2F sites in gene promoters (Supplementary Table S1). DNA occupancy was investigated by calculating the percentage enrichment of input for both the E2F1 ChIP and IgG controls from triplicate biological repeat experiments. In all cases, the presented figure displays mean enrichment from at least three biological repeat experiments with SD unless otherwise stated. The *CDC6* promoter was used as a positive control for E2F1 occupancy.

### RNA immunoprecipitation 

HCT116 WT E2F1 and E2F1 Cr cells were seeded into 15 cm dishes and treated with 1 μM PRMT5 inhibitor (T1-44) for 48 h where indicated. Media was removed from the dishes to allow UV cross-linking in an Amersham Life Sciences (Chicago, USA) ultra-violet crosslinker (400 mJ/cm^2^). Cells were then PBS washed and transferred to RNA immunoprecipitation (RIP) lysis buffer [50 mM Tris–HCl pH 7.4, 100 mM NaCl, 1% Igepal CA-630, 0.1% SDS, 0.5% Na-deoxycholate with protease inhibitors and RNaseOUT (Invitrogen, Waltham, USA)] for 30 min on ice. Samples were then sonicated with a probe sonifier (Branson digital sonifier, Branson Ultrasonics, Danbury, USA) at 25% amplitude for 10 cycles of 10 s on and 15 s off in ice. Samples were centrifuged at 13 000 rpm in a desktop centrifuge for 10 min prior to pre-clearing with 50 μl of protein A/G beads and 3 μg of non-specific IgG (as used for chromatin immunoprecipitation above) for 2 h at 4°C. At the same time, 4 μg of non-specific IgGs or specific antibodies [anti-HNRNPC (clone 4F4, Santa Cruz, Dallas, USA), anti-SC35 (sc-53518, Santa Cruz, Dallas, USA), and HNRNPH1 (ab10374, Abcam, Cambridge, UK)] were pre-bound to 50 μl of protein A/G beads at room temperature for 1 h in 100 μl NT2 buffer (50 mM Tris–HCl pH 7.4, 150 mM NaCl, 1 mM MgCl_2_, and 0.05% Igepal CA-630). Beads were washed three times in NT2 and then mixed with pre-cleared cell extracts overnight at 4°C. Beads were then washed five times in RIP lysis buffer and eluted in 150 μl of NT2 buffer containing 1% SDS and 1.2 mg/ml proteinase K at 55°C for 30 min. 500 μl of TRIzol was added to the supernatant and RNA was purified as per the manufacturer’s instructions. Isopropanol supplemented with GlycoBlue (Thermo Fisher, Waltham, USA) was used to precipitate RNA overnight at −20°C, and RNA was resuspended in 11 μl of nuclease-free H_2_O at 55°C for 10 min. The whole volume was then used to generate cDNA using Superscript III Reverse Transcriptase and random hexamers (Invitrogen, Waltham, USA).

QPCR was then performed in technical triplicate with Brilliant III Ultra-Fast SYBR green QPCR master mix on an AriaMx QPCR instrument (Agilent, Santa Clara, USA) using primers spanning the exon-intron boundaries of exons of interest (i.e. skipped exons and flanking exons) from spliced genes (Supplementary Table S1). RNA occupancy was investigated by calculating the percentage enrichment of input for both the specific antibody RIP and IgG controls from triplicate biological repeat experiments. In all cases, the presented figure displays SD unless otherwise stated.

### FACS analysis

Cells were harvested by trypsinization for FACS analysis after treatment with PRMT5 inhibitor (T1-44), hydroxyurea, etoposide, or double thymidine block as appropriate. Cells were washed in PBS prior to fixing with 70% ethanol in PBS at 4°C overnight. Cells were then stained with 40 μg/ml propidium iodide supplemented with 20 μg/ml RNase A in PBS for 1 h in the dark. DNA content of nuclei (cell cycle distribution and subG1) was analysed using a BD Accuri C6 flow cytometer with CFlow plus software (BD Biosciences, Franklin Lakes, USA). A total of 20 000 events were collected per sample.

### RNA sequencing

WT E2F1 HCT116 and E2F1 Cr HCT116 cells were treated with 1 µM concentration of PRMT5 inhibitor (T1-44) or DMSO as a negative control for 48 h. Total RNA from biological triplicate samples was isolated using TRIzol according to the manufacturer’s instructions. Alternatively, RNA isolated from three T1-44 treated or DMSO treated Colon26 mouse tumours *in situ* was used for RNA-seq analysis [24]. RNA-sequencing was performed by BGI Genomics (Shenzhen, China) as described previously [24].

WT pRb MCF7 and pRb Cr MCF7 cells were treated with 5 µM concentration of PRMT5 inhibitor (T1-44) or DMSO as a negative control for 72 h. mRNA was subsequently enriched from three biological replicates using the NEBNext Poly(A) mRNA magnetic isolation module (New England Biolabs, Ipswich, USA), as per the manufacturer’s instructions. cDNA libraries were made using the NEBNext ultra directional RNA library prep kit for Illumina (New England Biolabs, Ipswich, USA). Sequencing was carried out on an Illumina Next 500 Seq platform.

For RNA-seq performed on synchronized cells, total RNA from four biological repeat samples was isolated and analysed by BGI Genomics, as described briefly below: an Agilent 2100 Bioanalyzer (Agilent RNA 6000 Nano Kit, Santa Clara, USA) was used for RNA sample quality control purposes (RNA concentration, RIN value, 28S/18S, and the fragment length distribution). mRNAs were isolated from total RNA using oligo(dT)-based mRNA enrichment. mRNA was fragmented and reverse transcribed with random primer, prior to second strand synthesis with dUTP, end repair, and 3′ A-tailing. This was followed by adaptor ligation and digestion of U-labelled second strand template with uracil-DNA-glycosylase (UDG), prior to PCR amplification. Library quality control was performed before circularization and amplification to make DNA nanoballs for DNBseq (PE150 at ≥ 30 million reads per sample coverage).

Gene expression data have been deposited in the National Center for Biotechnology Information’s (NCBI) Gene expression Omnibus (GEO) and are accessible through GEO Series accession number GSE278461. RNA sequencing data from the HCT116 E2F1 Cr cells has previously been published [25] (under accession code: GSE142430). Colon26 tumour sample RNA-seq datasets have previously been published [24] and have been deposited to GEO under accession code GSE181401.

### RNAseq analysis

RNAseq raw data was processed with fastp software [36] to remove sequencing adaptors and low quality bases. The processed reads were aligned with human reference genome hg19 using the STAR package (v.020201) [37] with –quantMode Genecounts option to calculate the read counts. The differential expression analysis was performed with DESeq2 R package [38]. The consistency among RNA-seq replicates was assessed using standard approaches, including principal component analysis (PCA) and hierarchical clustering. All RNA-seq data and the derived count matrices are publicly available on GEO (GSE278461) and SRA. Genes were considered differentially expressed genes (DEGs) if the adjusted *P*-value, calculated using the Benjamini–Hochberg method in order to minimize the false discovery rate, was <0.05. We further filtered significant DEGs with fold change cut-offs, usually set to a 1.5-fold change in absolute expression level (equivalent to a log2 fold change of 0.58), unless otherwise indicated. The aligned data were also used for differential alternative splicing assessment with the rMATS program (v4.0.2) [39]. The FDR threshold for differential percent spliced in (PSI) values was chosen to be 0.05.

Alternatively, the cell cycle RNA-seq data was uploaded to the Galaxy web platform [40] at the public server at usegalaxy.org for analysis. Processed files were aligned with human reference genome hg38 using RNA STAR (Galaxy Version 2.7.11a) with per gene read counts option selected. Differential gene expression analysis was performed with DESeq2 (Galaxy version 2.11.40.8), whilst differential splicing analysis was performed with rMATS as described above.

Volcano plots of DEGs were generated in Galaxy with the volcano plot package (Galaxy version 0.0.5), whilst heatmaps were generated from the log2 fold change file output from DESeq2 using the heatmap2 package (Galaxy version 3.1.3.1) with the Canberra distance method for clustering. Heatmaps of differentially spliced events were generated from the delta PSI values output from the rMATS software.

### Gene annotation and analysis

Gene ontology analysis was performed on lists of DEGs (padj < 0.05, log2FC > 0.58) or on lists of differentially spliced genes (FDR < 0.05, ΔΨ > 0.1) that were observed to be uniquely regulated in wild-type E2F1 or wild-type pRb expressing cells treated with T1-44 (i.e. genes that were dependent on both E2F1 and PRMT5, or pRb and PRMT5), generated from each of the RNA-seq datasets. Analysis was performed using Metascape (https://metascape.org) [41] using GO biological processes or Reactome gene sets selected and a *P*-value cut-off for pathway and process enrichment set to 0.01.

### Measuring overlap between SRSF2/HNRNPC binding sites and alternative splicing events

Publicly available SRSF2 PAR-CLIP (GSE207643) and HNRNPC HITS-CLIP (GSE138726) and FLASH-CLIP (GSE94781) datasets were accessed from GEO.

For the SRSF2 data, processed supplementary BED files of SRSF2 binding cluster coordinates derived from biological duplicate samples were downloaded for HeLa and K562 cells. Data were further filtered by ReadCount ≥ 40 and any coordinates that did not intersect across biological repeats were removed. Bedtools (v2.31.1) [42] was then used to merge the remaining coordinates. For the HNRNPC HITS-CLIP data, processed supplementary BED files of crosslink site coordinates derived from biological triplicate samples in HeLa cells were downloaded. Overlapping crosslink sites were merged using Bedtools, and coordinates that intersected between biological repeats were collected and again merged. For the HNRNPC FLASH-CLIP data, processed BED files of crosslink site coordinates derived from biological duplicate samples in HEK293 cells were downloaded. Overlapping crosslink sites were merged using Bedtools, summing the number of PCR-duplicates in the score column. High confidence crosslink sites were filtered using a score ≥ 10, and coordinates that intersected between biological repeats were collected and again merged.

AS coordinates for significant differential splicing events output for each treatment in the HCT116 RNA-seq rMATS analysis were collected. Coordinates for both the target exon/intron and flanking exons were used. To identify overlaps between these splice coordinates and the SRSF2/HNRNPC CLIP sites, genomic intervals were expanded by 200 bp upstream and downstream using Bedtools slop prior to intersection with Bedtools intersect maintaining strand awareness. This extension accounts for the possibility that binding of a splicing factor near, but not directly on, a splice site can influence the splicing of adjacent exons and introns.

### Permutation analysis of CLIP binding site/AS coordinate overlaps

To assess the significance of overlap between CLIP coordinates and splicing associated genomic intervals, a strand specific permutation analysis was performed. CLIP coordinates were first classified as exonic or intronic using Bedtools intersect, based on overlap with merged exon and intron annotations from hg19, respectively. Coordinates overlapping both exonic and intronic regions were designated as ambiguous and randomly assigned to either category to preserve the original distribution of sites. Strand specific coordinate sets were generated by splitting features according to strand orientation. To maintain biological context during randomization, each strand specific set was independently shuffled using Bedtools shuffle with inclusion and exclusion criteria that restricted placement to the same genomic feature type (exon or intron) on the same chromosome and prevented overlap with the original CLIP coordinates. For each iteration, shuffled coordinates were combined and intersected with splicing associated regions as described above, to quantify random overlaps. This permutation was repeated 500 times in parallel [43], and the resulting counts were aggregated to generate a null distribution of expected overlaps. The observed number of overlaps between the original CLIP coordinates and splicing associated regions was then compared to this null distribution to assess enrichment significance (Monte Carlo *P*-value).

### E2F1 interactome analysis

20 × 15 cm dishes of HCT116 WT E2F1 cells were seeded and treated the next day with 1 μM PRMT5 inhibitor (T1-44) for 48 h. Dishes of untreated HCT116 E2F1 Cr cells were also included in the experiment to act as a negative control in the immunoprecipitation. Cells were harvested and an E2F1 immunoprecipitation experiment was performed as described above, using 5 μg of E2F1 KH-95 antibody (Santa Cruz, Dallas, USA). To ensure efficient enrichment of E2F1, the supernatant was immunoprecipitated a second time with a further 5 µg of anti-E2F1 antibody. Two rounds of elution were also performed on the bead material using Laemmli buffer (4% SDS, no glycerol, and no bromophenol blue). Eluates were combined and the volume reduced to <40 μl in an Eppendorf concentrator 5301 (Eppendorf, Hamburg, Germany). Sample volumes were normalized to 60 μl to dilute the SDS, prior to sample reduction, alkylation, and acidification. Proteins were digested in solution using S-Trap binding buffer (7:1) and 2 μg of trypsin on S-Trap micro columns (ProtiFi, Fairport, USA) as per the manufacturer’s instructions.

### Mass spectrometry data analysis

Tryptic peptides from three biological repeat experiments were analysed by liquid chromatography tandem mass spectrometry (LC-MS/MS) using the Orbitrap Fusion^™^ Lumos^™^ Tribrid^™^ Mass Spectrometer (Thermo Fisher, Waltham, USA) connected to Ultimate 3000 UHPLC system (Thermo Fisher, Waltham, USA). The fusion Lumos was operated in data-dependent mode, with advance peak detection (APD) enabled. Briefly, the samples were loaded on a trap column [PepMapC18 (Thermo Fisher, Waltham, USA); 300 µm × 5 mm, 5 µm particle size) and separated on a 50 cm EasySpray column (ES903, Thermo Fisher, Waltham, USA) using a linear gradient from 2% to 35% (v/v) solvent B in 5% DMSO in 60 min at 250 nl/min flow rate. The spectra were acquired with cycle times of 1 s. The MS1 spectra were acquired using the Orbitrap at 120 K resolution, with a scan time from 400 to 1500 *m*/*z*, an automatic gain control (AGC) target value of 4e5, and using s-lens RF30. The MS2 spectra were acquired using the Orbitrap at 30 K resolution, quadrupole isolation of 1.6, AGC target value of 5e4, and maximum injection time of 54 ms.

Samples were analysed in Fragpipe (v21.1) using the Label Free Quantitation with Match Between Runs (LFQ-MBR) workflow [44]. Files were searched against the reviewed human UniProt Swissprot database (downloaded November 2023, containing 20 426 sequences). MS Fragger settings were set to default, with strict trypsin selected (allowing two missed cleavages), carboamidomethylation (C) as fixed modification and oxidation (M) and acetylation (n-terminus) as variable modifications. Ion Quant parameters were left as default, and MBR was disabled.

Fragpipe combined-protein output was further analysed in Perseus (v1.6.2.2). Intensities were log 2 transformed before a filtering step to retain proteins present in all three biological replicates in at least one group was applied. Data were then normalized by median subtraction and missing values were imputed following normal distribution down shifted. A two-sample Student’s *t*-test combined with permutation-FDR multi-testing (5%) was performed. The mass spectrometry data have been deposited in ProteomeXchange consortium via the PRIDE partner repository with the dataset identifier PXD056505.

### Human E2F1 target gene promoter analysis

Gene promoter characterization was performed utilising bioinformatics tools present in UCSC Genome Browser (https://genome.ucsc.edu/ GRCh37/h19 assembly) and analysing ChIP-seq data for E2F tracks from the ENCODE project (http://genome.ucsc.edu/ENCODE/) for three cell lines (K562, MCF7, and HeLa). The ‘Transcription factor ChIP-seq clusters from ENCODE 3’, ‘Transcription factor ChIP-seq clusters from ENCODE with factorbook motifs’, ‘Transcription factor ChIP-seq peaks from ENCODE 3’, ‘Transcription factor ChIP-seq uniform peaks from ENCODE/Analysis,’ and ‘Transcription factor binding sites by ChIP-seq from ENCODE/Stanford/Yale/USC/Harvard’ track tools were used to display E2F1 ChIP-seq peaks or signal as appropriate. Genes were noted as potential direct E2F1 targets if ChIP-seq peaks were apparent within 1000-bp regions centred upon the annotated transcript start site (TSS) (i.e. 500 bp either side). Primers contained within these peak regions were designed for ChIP analysis (Supplementary Table S1).

### Statistical analysis

Statistical analyses were performed using two-tailed, unpaired Student’s *t*-test when only two samples were being compared, whilst one-way ANOVA using Sidak’s multiple comparisons test was performed in experiments involving multiple comparisons (with GraphPad Prism 10 Software, Dotmatics, Boston, USA). Data are shown as means with SD, unless otherwise indicated. *P* values lower than 0.05 were considered significant and are labelled using asterisks (*) for *P* < 0.05, (**) for *P* < 0.01, (***) for *P* < 0.001, and (****) for *P* ≤ 0.0001. The exact number of biological replicates is given in every figure legend.

## Results

### pRb–E2F pathway activity permits RNA splicing of E2F target genes

**Figure 1.**
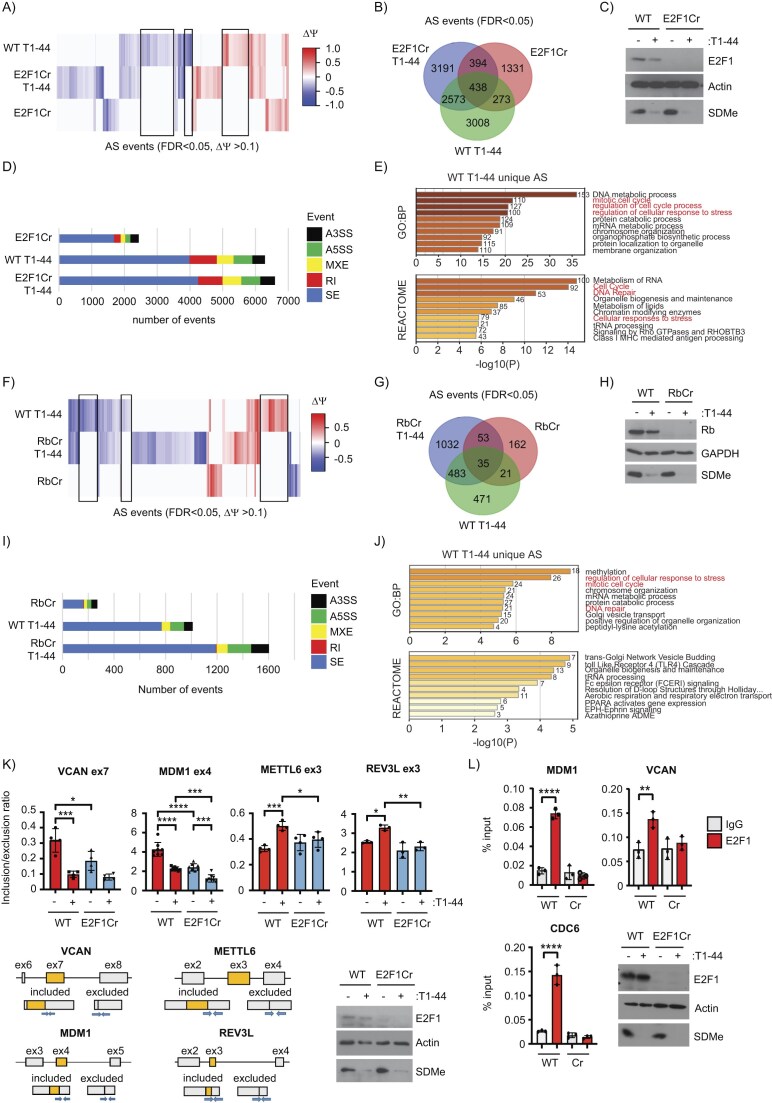
The pRb–E2F pathway regulates RNA splicing of E2F target genes. (**A**) Differential changes in splicing between WT and E2F1 Cr HCT116 cells, treated with DMSO or 1 μM T1-44 for 48 h are displayed as a heatmap of delta PSI values (ΔΨ, PSI) for all significant differential splicing events (FDR < 0.05, ΔΨ > 0.1). Each column of the heatmap represents delta PSI values of one splice event across all samples, as compared to the WT E2F1 HCT116 cells treated with DMSO (blue: reduced inclusion; red: increased inclusion). Data clustering used the Canberra distance method. Black boxes indicate splice events that uniquely occur upon T1-44 treatment, only in the presence of WT E2F1. These data were generated from three independent biological samples. (**B**) Venn diagram showing the overlap between statistically significant differential splicing events (FDR < 0.05) (AS) in each treatment, as compared to WT E2F1 HCT116 cells treated with DMSO. These data were generated from three independent biological samples. (**C**) A representative immunoblot displaying input protein levels of E2F1 and symmetric dimethylation (SDMe). Actin served as a loading control. (**D**) The bar chart displays the breakdown of statistically significant (FDR < 0.05) differential splicing events observed in each of the indicated treatments, as compared to WT E2F1 HCT116 cells treated with DMSO. SE, skipped/cassette exon; RI, retained intron; MXE, mutually exclusive exons; A5SS, alternative 5′ splice site; A3SS, alternative 3′ splice. These data were derived from the analysis in Fig. 1A and B. (**E**) Annotation of genes which undergo splice events that uniquely occur upon T1-44 treatment in the presence of WT E2F1 (see Fig. 1A). GO biological process (GO:BP) and Reactome gene sets were used for pathway analysis in Metascape. Biological terms connected with cell cycle, stress responses, and DNA damage are highlighted in red. The number of genes enriched in each category is displayed to the right of each bar. (**F**) Differential changes in splicing between WT and Rb Cr MCF7 cells, treated with DMSO or 1 μM T1-44 for 48 h are displayed as a heatmap of delta PSI values (ΔΨ, PSI) for all significant differential splicing events (FDR < 0.05, ΔΨ > 0.1). Each column of the heatmap represents delta PSI values of one splice event across all samples, as compared to the WT Rb MCF7 cells treated with DMSO (blue: reduced inclusion; red: increased inclusion). Data clustering used the Canberra distance method. Black boxes indicate splice events that uniquely occur upon T1-44 treatment, only in the presence of WT Rb. These data were generated from three independent biological samples. (**G**) Venn diagram showing the overlap between statistically significant differential splicing events (FDR < 0.05) (AS) in each treatment, as compared to WT Rb MCF7 cells treated with DMSO. These data were generated from three independent biological samples. (**H**) A representative immunoblot displaying input protein levels of Rb and SDMe. GAPDH served as a loading control. (**I**) The bar chart displays the breakdown of statistically significant (FDR < 0.05) differential splicing events observed in each of the indicated treatments, as compared to WT Rb MCF7 cells treated with DMSO. SE, skipped/cassette exon; RI, retained intron; MXE, mutually exclusive exons; A5SS, alternative 5′ splice site; A3SS, alternative 3′ splice. These data were derived from the analysis in Fig. 1F and G. (**J**) Annotation of genes which undergo splice events that uniquely occur upon T1-44 treatment in the presence of WT Rb (see Fig. 1F). GO biological process (GO:BP) and Reactome gene sets were used for pathway analysis in Metascape. Biological terms connected with cell cycle, stress responses, and DNA damage are highlighted in red. The number of genes enriched in each category is displayed to the right of each bar. (**K**) WT E2F1 and E2F1 Cr HCT116 cells treated for 48 h with 1 μM T1-44 or DMSO as indicated. An RT-PCR was performed to measure the inclusion of *VCAN* exon 7, *MDM1* exon 4, *METTL6* exon 3, or *REV3L* exon 3 in RNA transcripts from the cells. Displayed is the mean inclusion/exclusion ratio, with SD. Significance was calculated by ANOVA using Sidak’s multiple comparisons test. A diagram indicating the exon (boxed in grey) and intron (black lines) structure of each gene around the skipped exon (boxed in yellow) of interest is included. The splicing that gives rise to the exon included and excluded transcripts is also displayed, with specific primer pairs used in QPCR shown as blue arrows. A representative immunoblot is included to display input protein levels of E2F1 and SDMe. Actin was used as a loading control. (biological repeats: *n* = 4 for *VCAN* and *METTL6, n* = 3 for *REV3L*, and *n* = 8 for *MDM1*). See also Supplementary Fig. S1. (**L**) A ChIP assay performed on WT E2F1 or E2F1 Cr HCT116 cells. Recruitment of E2F1 to the promoter regions of *MDM1* and *VCAN* was tested. *CDC6* acted as a positive control. Displayed is the mean percentage enrichment of input, with SD. Significance was calculated by Student’s *t*-test between the indicated sample pairs. An immunoblot is included to display input protein levels of E2F1 and SDMe. Actin was used as a loading control (biological repeats: *n* = 3 for *MDM1, VCAN*, and *CDC6*).

Residue-specific arginine methylation by PRMT5 enables E2F1 to regulate many genes at the level of alternative RNA splicing (AS) [28]. To further elucidate the interplay between PRMT5 and E2F1 in the regulation of transcription and RNA splicing, we analysed an RNA-seq dataset from WT E2F1 and E2F1 CRISPR HCT116 cells (E2F1 Cr) treated with a highly selective PRMT5 inhibitor called T1-44 [24, 25]. We performed differential splicing analysis on the data using the rMATS algorithm [39], which indicated significant splice events were apparent in response to E2F1 expression or inhibition of PRMT5 activity (11 208 unique events across all treatments; FDR < 0.05) (Fig. 1A and B, and Supplementary Table S2); some splicing events were dependent on both E2F1 and PRMT5 since differential splicing only occurred in WT E2F1 expressing cells treated with T1-44 (Fig. 1A and B). This result suggests that for these splice events, E2F1 is a key target for PRMT5 activity. However, it was also evident that many splicing events occur at a significant level only in T1-44 treated E2F1 Cr cells, or only in E2F1 Cr cells treated with DMSO (Fig. 1A and B). This indicates that for the regulation of certain splice events E2F1 and PRMT5 must also act independently; for example, PRMT5 activity must be directed to other targets in E2F1 Cr cells. Effective inhibition of PRMT5 was monitored by immunoblotting with a pan-symmetric di-methyl arginine antibody (SDMe: Fig. 1C). These results show that each of PRMT5 and E2F1 can dictate which events are subjected to splicing control.

In the rMATs analysis, a variety of AS events were apparent, including alternative 3′ and 5′ splice site selection (A3SS and A5SS), mutually exclusive exons (MXE), retained introns (RI), and skipped exons (SE), the latter representing the most abundant AS event (Fig. 1D). Each type of splicing event was differentially regulated when a treatment condition was compared to the WT control DMSO treatment (Fig. 1D). For example, around 4000 SE were differentially spliced in WT cells after T1-44 treatment, whilst less than a 1000 RI, MXE, and A5SS/A3SS events each were observed under the same condition (Fig. 1D). We next selected splicing events that were dependent on both E2F1 and PRMT5 (boxed in black, Fig. 1A), and traced these events back to their source genes, to create a gene set that could be searched for enriched biological pathways using Metascape (https://metascape.org). This highlighted a number of biologically relevant terms connected with the cell cycle (such as the mitotic cell cycle and regulation of cell cycle process), the cellular response to stress and DNA repair (Fig. 1E).

We reasoned that the established functional relationship between pRb and E2F may also allow pRb to influence alternative splicing. We tested this idea by preparing a pRb Cr cell line and performing an RNA-seq analysis on WT and pRb Cr cells treated with T1-44. We analysed the dataset for differential splicing which identified a significant number of splice events, derived from numerous genes, under each condition, reflecting the influence of either pRb or PRMT5 (Fig. 1F–H, and Supplementary Table S3). As observed for the E2F1 splicing analysis, some splicing events were dependent on both pRb and PRMT5, since differential splicing only occurred in WT pRb expressing cells treated with T1-44, and not in the pRb Cr cells (Fig. 1F and G). It was noteworthy however that the splicing events seen to be influenced by pRb either alone or with PRMT5 inhibition, was considerably less than that seen for E2F1 (compare Fig. 1G to Fig. 1B). The majority of splicing events were SEs, although the other splicing events were also apparent (Fig. 1I). Selecting pRb- and PRMT5-dependent splicing events (boxed in black, Fig. 1F) allowed us to generate a gene set that was subsequently searched for enriched biological pathways with Metascape. Similar to the analysis performed for E2F1- and PRMT5-dependent spliced genes (Fig. 1E), the search highlighted biological terms connected with the cell cycle, the cellular response to stress and DNA repair (Fig. 1J).

We selected a small group of alternatively spliced (AS) genes from the rMATs data and analysed them further at the candidate gene level to confirm the influence of E2F1 and PRMT5. We chose *VCAN, MDM1, METTL6*, and *REV3L* because each gene had an exon where splicing was regulated in response to E2F1 and PRMT5 activity. In *VCAN*, alternative splicing of exon 7 was dependent on both E2F1 and PRMT5 activity (Fig. 1K), with a similar dependency apparent for exon 4 in *MDM1* (Fig. 1K). *METTL6* exon 3 and *REV3L* exon 3 alternative splicing was regulated by T1-44 treatment in an E2F1-dependent manner, since increased exon inclusion was only observed in treated WT cells and not E2F1 Cr cells (Fig. 1K). We next expanded our selection of candidate spliced genes to test a further 21 targets. Each spliced gene demonstrated dependency on either E2F1, PRMT5, or both, with some events observed to be regulated only upon T1-44 treatment in either WT E2F1 or E2F1 Cr cells (Supplementary Fig. S1). This observation was further confirmed for *MDM1* and *VCAN* across two independent E2F1 Cr clone cell lines (Supplementary Fig. S2A), in cells treated with the chemically distinct PRMT5 inhibitors, JNJ-64619178 and LLY-283 (Supplementary Fig. S2B), and in an independent E2F1 Cr cell line generated in PANC1 pancreatic cancer cells (Supplementary Fig. S2C). We also confirmed that *MDM1* and *VCAN* represented bone fide E2F1 target genes in HCT116 cells by monitoring E2F1 occupancy in their gene promoters by chromatin immunoprecipitation (ChIP). Similar to the known E2F1-target gene *CDC6*, E2F1 was detected in the chromatin of the promoter region of each spliced target gene in E2F1 expressing cells, in contrast to the E2F1 Cr cells where no E2F1 enrichment was observed (Fig. 1L).

To further examine the impact of other pRb-E2F pathway components on splicing of these candidate genes, we analysed the alternative splicing of *MDM1* exon 4 and *VCAN* exon 7 in pRb Cr and the double KO E2F1/pRb Cr cell lines (Supplementary Fig. S3A) or in cells treated with DP1 siRNA (Supplementary Fig. S3B). The DP1 protein functions as the heterodimeric partner of E2F subunits, including E2F1, in mammalian cells and it is the heterodimeric E2F1/DP1 complex which physically associates with pRb [45]. Under conditions of pRb loss, or DP1 siRNA treatment, a similar effect on splicing was observed as reported for E2F1 Cr cells, namely both pRb and DP1 were positive regulators of exon inclusion (Supplementary Fig. S3A–C). Indeed, manipulating multiple pRb–E2F pathway components at once (i.e. knockout of both pRb and E2F1 in the same Cr cell line, Supplementary Fig. S3A; or treatment with DP1 siRNA in the E2F1 Cr or pRb Cr cells, Supplementary Fig. S3B) had no further impact on splicing of the *MDM1* or *VCAN* exon, suggesting that pRb, E2F1 and DP1 act through a common pathway to regulate alternative splicing of these genes. The impact of PRMT5 inhibition on splicing in these assays was also completely abrogated or quantitatively reduced in cells lacking E2F1, pRb, or DP1 (Supplementary Fig. S3A and B), suggesting that the influence of PRMT5 activity requires the functional integrity of the pRb–E2F pathway. Generally, these results support the role of pRb, E2F1, DP1, and PRMT5 in regulating AS of E2F target genes. They also suggest that each distinct pathway component can have a quantitative and qualitative impact on the network of genes regulated by alternative splicing.

### pRb–E2F pathway regulates distinct gene sets through RNA splicing and transcription

**Figure 2.**
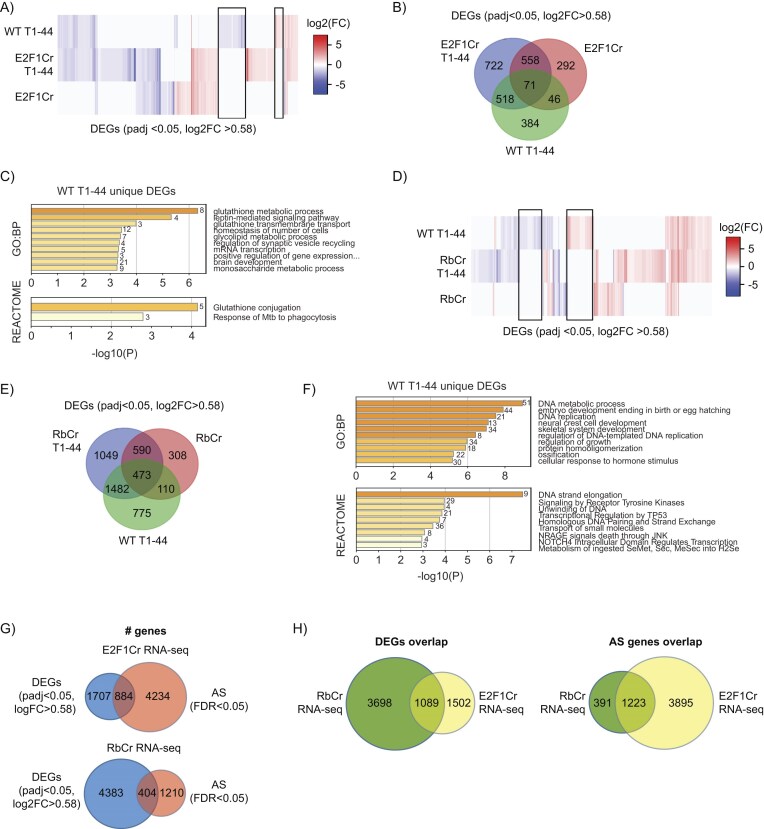
The pRb–E2F pathway regulates separate transcription and splicing gene networks. (**A**) Differential gene expression between WT and E2F1 Cr HCT116 cells, treated with DMSO or 1 μM T1-44 for 48 h, are displayed as a heatmap of log2 fold change (log2FC) values for all significant DEGs (padj < 0.05, log2FC > 0.58). Each column of the heatmap represents log2FC values of one DEG across all samples, as compared to the WT E2F1 HCT116 cells treated with DMSO (blue: reduced expression; red: increased expression). Data clustering used the Canberra distance method. Black boxes indicate DEGs that uniquely occur upon T1-44 treatment, only in the presence of WT E2F1. These data were generated from three independent biological samples. See also Supplementary Fig. S3D. (**B**) A Venn diagram showing the overlap between statistically significant DEGs (padj < 0.05, log2FC > 0.58) in each treatment, as compared to WT E2F1 HCT116 cells treated with DMSO. See also Supplementary Fig. S3D. (**C**) Annotation of genes which only undergo differential expression upon T1-44 treatment in the presence of WT E2F1 (see Fig. 2A). GO biological process (GO:BP) and Reactome gene sets were used for pathway analysis in Metascape. The number of genes enriched in each category is displayed to the right of each bar. (**D**) Differential gene expression between WT and Rb Cr MCF7 cells, treated with DMSO or 1 μM T1-44 for 48 h, are displayed as a heatmap of log2 fold change (log2FC) values for all significant DEGs (padj < 0.05, log2FC > 0.58). Each column of the heatmap represents log2FC values of one DEG across all samples, as compared to the WT Rb MCF7 cells treated with DMSO (blue: reduced expression; red: increased expression). Data clustering used the Canberra distance method. Black boxes indicate DEGs that uniquely occur upon T1-44 treatment, only in the presence of WT Rb. These data were generated from three independent biological samples. See also Supplementary Fig. S3E. (**E**) A Venn diagram showing the overlap between statistically significant DEGs (padj < 0.05, log2FC > 0.58) (DEGs) in each treatment, as compared to WT Rb MCF7 cells treated with DMSO. See also Supplementary Fig. S3E. (**F**) Annotation of genes which only undergo differential expression upon T1-44 treatment in the presence of WT Rb (see Fig. 2D). GO biological process (GO:BP) and Reactome gene sets were used for pathway analysis in Metascape. The number of genes enriched in each category is displayed to the right of each bar. (**G**) Venn diagrams displaying the overlap of total genes from the E2F1 Cr and Rb Cr RNA-seq datasets that score either as significantly differentially expressed (DEGs: padj < 0.05; log2FC > 0.58), significantly differentially spliced (AS: FDR < 0.05), or fall into both categories. These data were derived from the analyses in Figs 1 and 2. (**H**) Venn diagrams showing the overlap between the total list of statistically significant DEGs (padj < 0.05, log2FC > 0.58), or the total list of statistically significant differentially spliced genes (AS: FDR < 0.05) in each of the indicated RNA-sequencing datasets, as compared to their corresponding wild-type cell lines.

To examine the relationship between genes regulated by pRb–E2F at the level of alternative RNA splicing, with those that are regulated at the level of transcription, we examined DEGs namely transcription targets between treatment groups. We identified a large number of condition-specific DEGs that were dependent on E2F1, PRMT5, or a combination of the two (since some genes were only differentially expressed in WT cells treated with T1-44, and not in E2F1 Cr-treated cells) (Fig. 2A and B, Supplementary Fig. S3D and Supplementary Table S2). From the analysis, we generated a set of genes that were uniquely regulated in T1-44 treated, WT E2F1 expressing cells, namely where the expression is dependent on E2F1 and PRMT5 (Fig. 2A, boxed in black), which we then used to search for enriched biological pathways using Metascape. This analysis highlighted a number of biological terms connected with transcription, metabolic processes, and gene expression (Fig. 2C), as has been observed previously [25]. These biological terms are distinct from those identified for the E2F1- and PRMT5-dependent AS gene sets (Fig. 1E), suggesting that distinct biological pathways are impacted by genes controlled at the level of transcription, or AS regulation, by E2F1 and PRMT5.

We performed a similar examination on the pRb Cr cell line RNA-seq dataset, where a DEG analysis identified specific DEGs dependent on pRb and PRMT5, and a combination of the two (Fig. 2D and E, Supplementary Fig. S3E and Supplementary Table S3). Using genes that were uniquely regulated in T1-44 treated, WT pRb expressing cells (Fig. 2D, boxed in black) as the selected gene set, we identified a number of biological terms connected with transcriptional processes, metabolism, and DNA replication (Fig. 2F). Once more, these terms were distinct from those identified for the pRb- and PRMT5-dependent AS gene sets (Fig. 1J), supporting the conclusion that distinct biological pathways are regulated at the level of transcription and alternative splicing by pRb–E2F and PRMT5.

To test this hypothesis further, we examined the overlap in the RNA-seq datasets between genes regulated at the transcriptional level by E2F1, pRb, and PRMT5, as compared to those genes impacted by alternative splicing (Fig. 2G). It was apparent that the majority of genes (87% in the E2F1 dataset, and 93% in the pRb dataset) were present either in the DEG or AS gene sets (Fig. 2G), with a smaller percentage of genes overlapping in both groups. These results support the conclusion that E2F1 and pRb regulate distinct gene sets through transcription or alternative splicing. Interestingly, the non-overlapping set of AS genes influenced by E2F1 and PRMT5 (total 4234) was much larger than the non-overlapping set of DEG targets (total 1707), suggesting that E2F1 and PRMT5 are more prominent regulators of AS events (Fig. 2G). This is in contrast to pRb, which was seen to be more relevant for the transcription gene set, where the DEG target gene set (4383) was much larger than the set of AS genes (1210) (Venns shown in Fig. 2G).

When the list of genes from the two RNA-seq datasets (derived from E2F1 or pRb Cr cells) were compared, a large set of shared genes were consistently identified in the two analyses (DEGs: 1089; AS: 1223), scoring as either shared DEGs or shared AS genes (left venn, or right venn of Fig. 2H, respectively). However, it was once more apparent that a much larger number of AS genes were uniquely identified in the E2F1 dataset than in the Rb dataset (E2F1 = 3895 compared to pRb = 391), whilst the opposite was true for DEGs (E2F1 = 1502 compared to pRb = 3698) (Fig. 2H). These results therefore suggest the following conclusions. Firstly, that E2F1 primarily acts to regulate genes at the level of AS, whilst pRb appears more relevant for transcriptional control of target genes. Secondly, whilst each component of the pRb–E2F pathway can contribute to both the transcription and splicing of target genes, many genes exhibit a preference for control by either pRb or E2F1.

### Alternative splicing of E2F target genes occurs in tumours *in situ*

**Figure 3.**
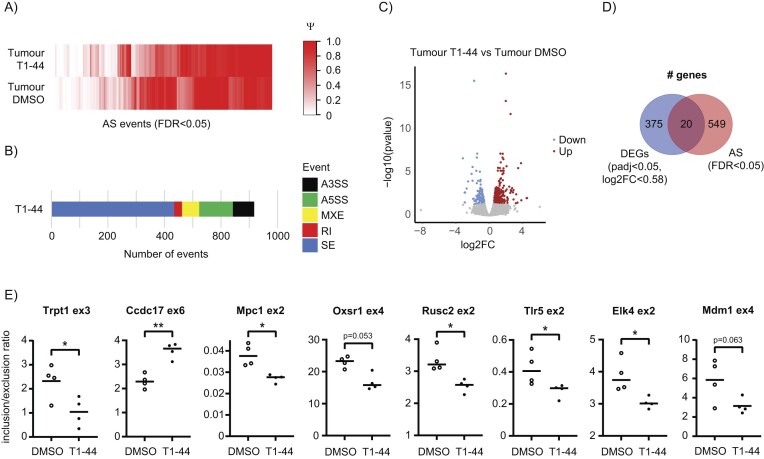
PRMT5 regulates pRb–E2F transcriptional and splicing networks *in vivo*. (**A**) Differential changes in splicing between Colon26 tumours isolated from mice treated with T1-44, or treated with DMSO, displayed as a heatmap of PSI values (Ψ, PSI) for all significant differential splicing events (FDR < 0.05). Each column of the heatmap represents PSI values of one splice event across all samples. Data clustering used the Canberra distance method. These data were generated from four mice per treatment group. (**B**) The bar chart displays the breakdown of statistically significant (FDR < 0.05) differential splicing events observed in Colon26 tumours from mice treated with T1-44 as compared to tumours from mice treated with DMSO. SE, skipped/cassette exon; RI, retained intron; MXE, mutually exclusive exons; A5SS, alternative 5′ splice site; A3SS, alternative 3′ splice. These data were derived from the analysis in Fig. 3A. (**C**) A volcano plot displaying values of log2 fold change (log2FC) and –log10 *P*-values for DEGs identified between Colon26 tumours from mice treated with T1-44 as compared to tumours from mice treated with DMSO. Red colour represents genes upregulated by T1-44 treatment, whilst blue colour represents genes downregulated by T1-44 treatment. Grey colour represents genes that fell below the fold change or statistical cut-off applied (padj < 0.05, log2FC > 0.58). These data were derived from the same RNA-seq analysis used to generate Fig. 3A. (**D**) A venn diagram displaying the overlap of total genes from the Colon26 tumour RNA-seq that score either as significantly differentially expressed (DEGs: padj < 0.05, log2FC > 0.58), significantly differentially spliced (AS: FDR < 0.05), or fall into both categories. These data were derived from the analysis in Fig. 3A and C. (**E**) RNA extracted from Colon26 tumours of mice treated with T1-44 or DMSO was used in an RT-PCR experiment to measure the inclusion of the indicated exons in RNA transcripts. Displayed is the mean inclusion/exclusion ratio for each gene, with individual data points displayed. Significance was calculated by Student’s *t*-test (*n* = 4 mice from each treatment group).

So far, our experiments have focussed on analysing gene expression in cells *in vitro*. It was of interest therefore to address whether similar effects occurred in a tumour model. For this, we performed an experiment using the murine Colon26 colorectal cancer syngeneic model, in which effective concentrations for T1-44 that caused a significant delay in tumour growth have previously been documented [24]. Two groups of mice were analysed; one control group and the other treated with compound T1-44, and RNA-seq was performed on tumours taken from both groups of mice (Fig. 3A–D).

Both AS and DEG target genes were apparent when tumours from T1-44 treated mice were compared to untreated tumours (Fig. 3A and C and Supplementary Table S4), and again the overlap of genes identified as DEGs with those genes identified as AS was minimal (∼2.1%) (Fig. 3D). Furthermore, when the equivalent gene selective splicing events were investigated at the candidate gene level, similar AS changes were observed. For example, inclusion of *Mdm1* exon 4 and *Trpt1* exon 3 was reduced in tumours treated with T1-44 (Fig. 3E), as was observed in the human cell lines (Fig. 1K, and Supplementary Fig. S1A and D), though statistical significance was not always achieved. We also selected additional splice events identified in the tumour RNA-seq analysis and tested their behaviour in T1-44 treated and untreated samples. In each case, the splicing event behaved as anticipated, displaying either an increased (*Ccdc17* exon 6) or decreased exon inclusion (*Mpc1* exon 2, *Oxsr1* exon 4, *Rusc2* exon 2, *Tlr5* exon 2, and *Elk4* exon 2) in response to T1-44 treatment (Fig. 3E). We conclude that alternative splicing and transcription networks of E2F target genes are regulated *in situ* in tumours.

### Alternative splicing of E2F target genes is regulated during the cell cycle

**Figure 4.**
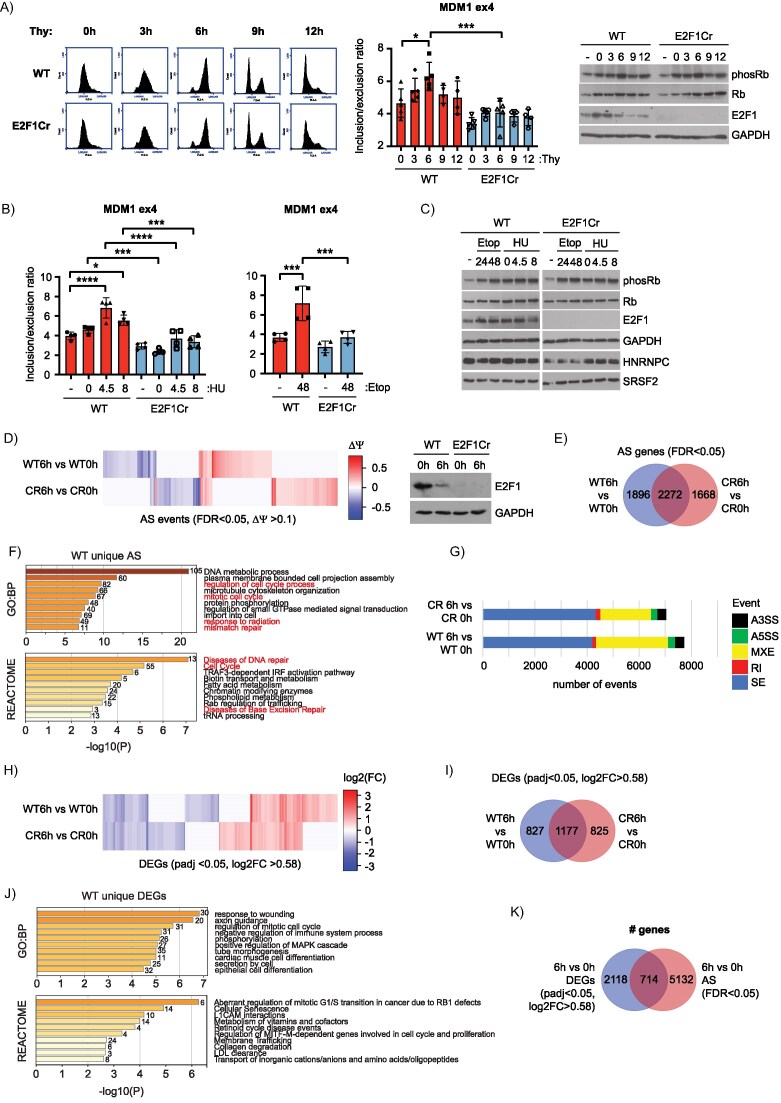
Cell cycle regulated alternative splicing and transcriptional events dependent on E2F1. (**A**) On the left, a representative flow cytometry profile for wild-type (WT) E2F1 and E2F1 Cr HCT116 cells synchronized at the G1/S boundary with a double thymidine block (0 h timepoint), or released from the block to progress through S phase (3 h) into G2/M (6 h), and back into a subsequent G1 (9 and 12 h) are displayed. An RT-PCR was performed at each timepoint to measure the inclusion of *MDM1* exon 4 in RNA transcripts. Displayed is the mean inclusion/exclusion ratio, with SD. Significance was calculated by ANOVA using Sidak’s multiple comparisons test. A representative immunoblot is included to display input protein levels of Rb, phosphorylated Rb and E2F1. GAPDH was used as a loading control (*n* = 4 biological repeats). See also Supplementary Fig. S3G. (**B**) WT E2F1 and E2F1 Cr HCT116 cells were treated with 1 mM hydroxyurea (HU) for 24 h to synchronize cells in early S phase. Hydroxyurea was then washed out and cells were allowed to progress through S phase for the indicated number of hours. Alternatively, cells were treated with 20 μM etoposide (Etop) for 48 h where indicated, and an RT-PCR was performed to measure the inclusion of *MDM1* exon 4 in RNA transcripts. Displayed is the mean inclusion/exclusion ratio, with SD. Significance was calculated by ANOVA using Sidak’s multiple comparisons test (*n* = 4 biological repeats). See also Supplementary Fig. S4B–D. (**C**) A representative immunoblot to display input protein levels of Rb, phosphorylated Rb, E2F1, HNRNPC, and SRSF2 for the experiments described in Fig. 4B. GAPDH was used as a loading control. (**D**) Differential changes in splicing between WT E2F1 and E2F1 Cr HCT116 cells synchronized in G1/S (0 h) or G2/M (6 h) are displayed as a heatmap of delta PSI values (ΔΨ, PSI) for all significant differential splicing events (FDR < 0.05, ΔΨ > 0.1). Each column of the heatmap represents delta PSI values of one splice event in cells at 6 h, as compared to the same cells at 0 h (blue: reduced inclusion; red: increased inclusion). Data clustering used the Canberra distance method. These data were generated from four independent biological samples. A representative immunoblot is included to display the input protein levels for E2F1. GAPDH was used as a loading control. (**E**) Venn diagram showing the overlap between statistically significant differentially spliced genes (AS: FDR < 0.05) between the 6 h (G2/M) and 0 h (G1/S) timepoints, in WT E2F1 and E2F1 Cr HCT116 cells. These data were derived from the analysis in Fig. 4D. (**F**) Annotation of genes which undergo splice events that uniquely occur in WT E2F1 cells between the 6 and 0 h timepoints (see Fig. 4D). GO biological process (GO:BP) and Reactome gene sets were used for pathway analysis in Metascape. Biological terms connected with cell cycle and DNA damage/repair are highlighted in red. The number of genes enriched in each category is displayed to the right of each bar. (**G**) The bar charts display the breakdown of statistically significant (FDR < 0.05) differential splicing events observed between each of the indicated treatments. SE, skipped/cassette exon; RI, retained intron; MXE, mutually exclusive exons; A5SS, alternative 5′ splice site; A3SS, alternative 3′ splice. These data were derived from the analysis in Fig. 4D. (**H**) Differential changes in gene expression between WT E2F1 and E2F1 Cr HCT116 cells synchronized in G1/S (0 h) or G2/M (6 h) are displayed as a heatmap of log2 fold change (log2FC) values for all significant DEGs (padj < 0.05, log2FC > 0.58). Each column of the heatmap represents log2FC values of one DEG in cells at 6 h, as compared to the same cells at 0 h (blue: reduced expression; red: increased expression). Data clustering used the Canberra distance method. These data were generated from four independent biological samples. See also Supplementary Fig. S5D. (**I**) Venn diagram showing the overlap between statistically significant DEGs (padj < 0.05, log2FC > 0.58) between the 6 h (G2/M) and 0 h (G1/S) timepoints, in WT E2F1 and E2F1 Cr HCT116 cells. These data were derived from the analysis in Fig. 4H. (**J**) Annotation of genes which undergo differential expression only in WT E2F1 cells between the 6 and 0 h timepoints (see Fig. 3H). GO biological process (GO:BP) and Reactome gene sets were used for pathway analysis in Metascape. The number of genes enriched in each category is displayed to the right of each bar. (**K**) Venn diagram displaying the overlap of total genes from the RNA-seq experiment in Fig. 4D and H, that score either as significantly differentially expressed (DEGs: padj < 0.05, log2FC > 0.58), significantly differentially spliced (AS: FDR < 0.05), or fall into both categories.

Given the enrichment of cell cycle, stress response, and DNA repair related biological pathways in the AS gene lists (Fig. 1E and J), and the well documented role of the pRb–E2F pathway in these processes [5, 17, 20], we decided to examine whether E2F1 and PRMT5 dependent splicing was regulated during the cell cycle or in response to DNA damage. To address this possibility we firstly synchronized WT E2F1 or E2F1 Cr HCT116 cells at the G1/S boundary using a double thymidine block [46] (0 h timepoint), which was then released so that cells could be followed through a single cell cycle and back into the next G1 (12h timepoint) (Fig. 4A and Supplementary Fig. S3G). Since E2F target genes are known to be under cell cycle control at the transcriptional level [5, 47], we confirmed their regulation in our synchronized cells by measuring a number of established targets. *Cyclin E1* and *E2* mRNA (*CCNE1* and *CCNE2*) peak in mid-G_1_ to early S phase and decline upon mitotic division [48], whilst *cyclin A* mRNA (*CCNA2*) expression peaks as cells progress through G2 phase into mitosis [49]. Similar effects were observed in our synchronized cells for the expression of each gene (Supplementary Fig. S4A). *E2F1* levels themselves declined as cells entered G2, consistent with the previously described turnover of E2F1 in S phase [50]. As expected, RNA levels for *E2F1* were low in E2F1 Cr cells throughout the cell cycle (Supplementary Fig. S4A). Having established that our synchronized cells were behaving as expected, we next tested the impact of the cell cycle on AS by examining splicing of *MDM1* exon 4. Inclusion of *MDM1* exon 4 increased as cells moved from G1/S into G2/M phase (0 h to 6 h timepoints), before declining as cells approached the next G1 (12 h timepoint) (Fig. 4A). This was dependent on E2F1, since the periodic activation of exon 4 AS was absent in the E2F1 Cr cells (Fig. 4A).

We next examined the DNA damage response, in which E2F1 has an established role [17, 20]. In cells exposed to DNA damaging agents, like etoposide (arrests cells in G2 by causing DNA double strand breaks, Supplementary Fig. S4B) or hydroxyurea (arrests cells at G1/S boundary and induces replication stress, Supplementary Fig. S4C), E2F1 is stabilized which leads to cell cycle arrest and apoptosis [51–53]. Again, *MDM1* AS was impacted by both treatments, and this was dependent on E2F1 since increased inclusion of exon 4 in response to hydroxyurea or etoposide was only apparent in WT E2F1 cells (Fig. 4B and C). As expected, *E2F1* gene expression increased in response to hydroxyurea and etoposide (Supplementary Fig. S4B and C), whilst *CCNA2* expression peaked as cells moved into G2/M phase (either as a result of etoposide treatment, Supplementary Fig. S4B; or 8 h after release from a hydroxyurea-induced G1 phase arrest, Supplementary Fig. S4C). Other spliced gene targets, including *TRPT1* and *SORBS1*, also demonstrated AS events that were regulated in response to cellular stress (Supplementary Fig. S4D).

We also investigated the role of PRMT5 in regulating AS by combining etoposide or hydroxyurea treatment with compound T1-44 (Supplementary Fig. S5A–C). We noted that the increased inclusion of *MDM1* exon 4 in WT E2F1 cells treated with hydroxyurea (Supplementary Fig. S5Aii) or etoposide (Supplementary Fig. S5Bii) was absent or quantitatively reduced in cells co-treated with T1-44, or in cells lacking E2F1 expression, suggesting that the AS regulation of this gene is not only influenced by E2F1 but also requires PRMT5 activity. These results indicate that alternative splicing of E2F target genes can occur in diverse cellular contexts and exhibits dependency on E2F1 and PRMT5.

### Global analysis of cell cycle regulated RNA splicing

Given the results suggesting that AS of candidate genes is under cell cycle control (Fig. 4A–C), we next addressed whether global AS events are regulated during cell cycle progression by performing RNA-seq on HCT116 WT E2F1 and E2F1 Cr cells synchronized by double thymidine block. We compared the 0 and 6 h timepoints post thymidine block, since these were the conditions showing the greatest impact on *MDM1* splicing (Fig. 4A). We analysed the RNA-seq datasets using rMATS and observed a remarkably high number of significant AS events regulated between 0 and 6 h, when most cells had progressed through S phase into G2/M phase. By flow cytometry, the cell cycle profile in thymidine-released cells was similar between WT and E2F1 Cr cells (Fig. 4A and Supplementary Fig. S4A).

To assess how many AS events were dependent on E2F1 activity we compared the rMATS analysis of WT cells with the E2F1 Cr cell data (Fig. 4D–G), and observed many events that were E2F1-dependent (Fig. 4D and E, and Supplementary Table S5); 4168 genes were subject to AS during cell cycle progression in WT E2F1 cells, with 1896 of these genes being spliced in an E2F1 dependent manner (Fig. 4D and E). Interestingly, 1668 genes underwent cell cycle dependent AS only in E2F1 Cr cells, suggesting that in some cases the presence of E2F1 suppresses AS (Fig. 4D and E). Examining enriched biological pathways in the E2F1-dependent AS gene set identified terms related to DNA repair and cell cycle transitions, when comparing between the 0 and 6 h timepoint (Fig. 4F). As with our previous analysis in E2F1 Cr cells (Fig. 1E), this suggests that regulation of global AS events by E2F1 is relevant to cell cycle control and the response to DNA damage. Skipped exons (SE) were the predominant form of splicing observed during cell cycle progression, though mutually exclusive exons (MXE) also represented an abundant splice type, with E2F1 Cr cells demonstrating a reduced number of MXEs as compared to WT cells (Fig. 4G).

We also examined the synchronized RNA-seq datasets for differential gene expression (Fig. 4H–J). By inspecting the RNA-seq dataset of WT E2F1 cells, a large number of genes (2004) scored as differentially expressed from 6 h post thymidine release (Fig. 4H and I, Supplementary Fig. S5D and Supplementary Table S5), when most cells had progressed through S phase into G2/M phase (Supplementary Fig. S4A). To test how many DEGs were dependent on E2F1 activity we performed the same analysis on the E2F1 Cr cell data (Fig. 4H and I, and Supplementary Fig. S5D and Supplementary Table S5). A set of non-overlapping DEGs were specifically regulated in the WT or E2F1 Cr cells between 0 and 6 h (827 or 825, respectively) with a larger group of overlapping DEGs (1177) (Fig. 4H and I). We conclude therefore that whilst many genes are differentially transcribed as cells progress from G1 through the cell cycle into G2/M phase, E2F1 is specifically responsible for regulating the transcription of a large subset of these genes, namely 1652.

Examining enriched biological pathways in this E2F1-dependent DEG gene set revealed terms related to cell cycle progression, cell differentiation, and cell signalling pathways (Fig. 4J). These biological terms are distinct from the DNA repair terms identified in the AS gene sets (Fig. 4F), suggesting that the separate transcription and splicing gene networks influence distinct cellular processes. In support of this idea, most of the genes that scored as AS targets were mutually exclusive with genes scoring as DEGs (Fig. 4K), highlighting the distinct gene networks that are DEG or AS targets.

**Figure 5.**
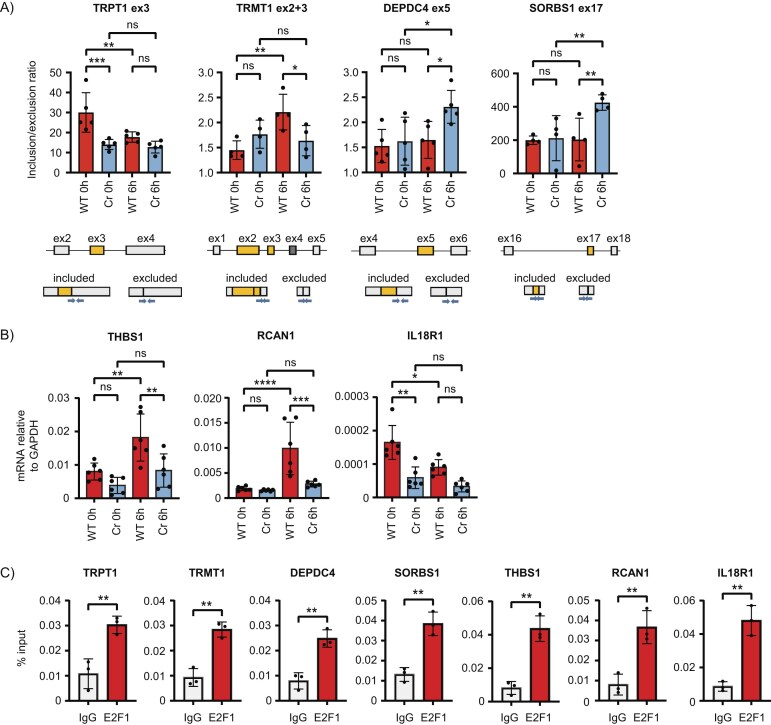
Confirmation of cell cycle regulated alternative splicing and transcriptional events in cells. (**A**) RNA from WT E2F1 and E2F1 Cr cells either in G1/S (0 h) or G2/M (6 h) was used in an RT-PCR experiment to monitor the inclusion of exon 3 in *TRPT1*, exons 2 and 3 in *TRMT1*, exon 5 in *DEPDC4*, and exon 17 in *SORBS1*, each identified as being significantly differentially spliced in the analysis performed in Fig. 4D. Displayed is the mean inclusion/exclusion ratio with SD. A diagram indicating the exon (boxed in grey) and intron (black lines) structure of each gene around the skipped exon (boxed in yellow) of interest is included. The splicing that gives rise to the exon included and excluded transcripts is also displayed, with specific primer pairs used in QPCR shown as blue arrows. Significance was calculated by ANOVA using Sidak’s multiple comparisons test. (biological repeats: *n* = 4 for *TRMT1* ex2 + 3 and *SORBS1* ex17, *n* = 5 for *TRPT1* exon 3 and *DEPDC4* exon 5). (**B**) RNA from WT E2F1 and E2F1 Cr cells either in G1/S (0 h) or G2/M (6 h) was used in an RT-PCR experiment to monitor the expression of genes (*THBS1, RCAN1* and *IL18R1*) identified as significantly differentially expressed in the RNA-sequencing experiment performed in Fig. 4H. Displayed is the mean mRNA expression relative to the *GAPDH* internal calibrator, with SD. Significance was calculated by ANOVA using Sidak’s multiple comparisons test (*n* = 6 biological repeats). (**C**) A ChIP assay performed on wild-type E2F1 HCT116 cells. Recruitment of E2F1 to the promoter regions of the indicated differentially spliced genes (*TRPT1, TRMT1, DEPDC4*, and *SORBS1*) and DEGs (*THBS1, RCAN1*, and *IL18R1*) was tested. Displayed is the mean percentage enrichment of input, with SD. Significance was calculated by Student’s *t*-test between the indicated sample pairs (*n* = 3 biological repeats).

To confirm the results from the RNA-seq analysis, we selected a small set of genes with exons that were AS and analysed them further. For example, exon 3 of *TRPT1* showed elevated inclusion levels in WT E2F1 cells at the 0 h timepoint, with more exon skipping occurring as cells progressed to the 6 h timepoint (Fig. 5A). The opposite was observed for exons 2 and 3 of *TRMT1*, with exon inclusion increasing at 6 hours post thymidine release (Fig. 5A). This cell cycle dependency was not observed in the E2F1 Cr cells, which demonstrated similar levels of *TRPT1* and *TRMT1* exon inclusion at both timepoints (Fig. 5A). For the splicing of some genes, cell cycle dependent changes in exon inclusion were only apparent in E2F1 Cr cells, with WT cells displaying no significant cell cycle dependent changes in splicing. For example, the inclusion of exon 5 of *DEPDC4* and exon 17 of *SORBS1* increased at the 6 h timepoint only in the absence of E2F1 (Fig. 5A). We also observed at the level of ChIP analysis that these spliced genes were all E2F1 target genes (Fig. 5C).

We performed a similar candidate level gene analysis on cell cycle regulated DEGs, where we identified a number of genes that are under cell cycle control and exhibit E2F1 dependency. For example, *THBS1, RCAN1*, and *IL18R1* had distinguishable transcription profiles, with *THBS1* and *RCAN1* peaking at 6 h as cells moved through G2, whereas *IL18R1* was highest at 0 hours in G1 cells (Fig. 5B). In each case, there was a clear difference between the transcription in WT and E2F1 Cr cells (Fig. 5B). The promoter regions of all three genes were subsequently identified by ChIP to be specific targets for E2F1 (Fig. 5C). These results thus describe examples of candidate E2F target genes that are under splicing or transcriptional control by the E2F pathway.

### Splicing factors associate with the E2F complex

**Figure 6.**
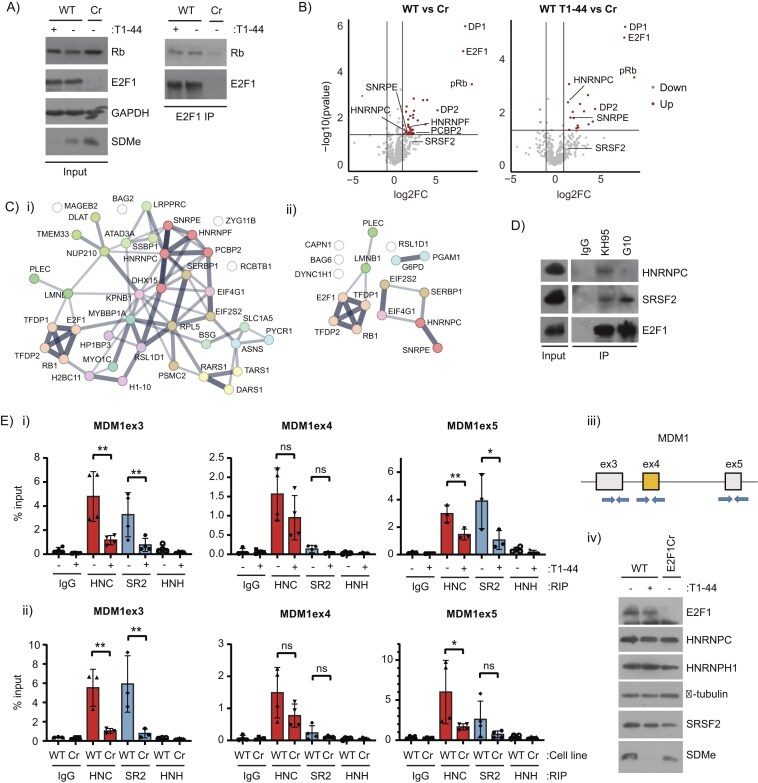
RNA splicing factors associate with the E2F complex and are recruited to AS genes. (**A**) A representative immunoblot displaying the immunoprecipitation of E2F1 from WT HCT116 cells treated with 1 μM T1-44 or DMSO for 48 h where indicated. E2F1 Cr HCT116 cells were used in the E2F1 immunoprecipitation as a control. Rb is displayed on the immunoblots as a known interactor for E2F1, and SDMe is used as a control for T1-44 activity. GAPDH is presented as a loading control. The immunoprecipitated material was used in a downstream mass spectrometry analysis of the E2F1 interactome (*n* = 3 bioligical repeats). (**B**) Volcano plots displaying log2 fold change (log2FC) and –log10 *P*-values for relative intensities of interacting proteins in E2F1 immunoprecipitates performed in T1-44 treated or untreated WT cells, as compared to immunoprecipitations performed in the control E2F1 Cr cell line. Red colour represents interacting proteins enriched in the WT E2F1 immunoprecipitates, whilst blue colour represents under-enriched proteins. Grey colour represents proteins that fell below the fold change or statistical cut-off applied (*P* < 0.05, log2FC > 1). Marked on the figure are known E2F1 interacting proteins (Rb, E2F1, DP1, and DP2) and proteins implicated in RNA splicing and processing. These data were derived from the mass spectrometry experiment performed in Fig. 6A (*n* = 3 biological repeats). (**C**) Functional protein association networks for proteins identified as E2F1 interactors from mass spectrometry analysis performed on DMSO treated (i), or T1-44 treated cells (ii) were generated using STRING. Proteins that were enriched in E2F1 immunoprecipitations performed in WT cells, as compared to E2F1 Cr cells (fold change > 2), at a statistically significant level (*P* < 0.05) were included. The edges indicate both functional and physical protein associations, with line thickness indicating the strength of data support. MCL clustering of proteins was performed. This figure was generated using the data from Fig. 6B. (**D**) An immunoprecipitation experiment was performed in WT E2F1 HCT116 cells using the indicated antibodies against E2F1 (KH95 and G10 antibodies) or control IgG. Interacting SRSF2 and HNRNPC was detected using specific antibodies. Input protein levels are also displayed (*n* = 2 bioligical repeats). See also Supplementary Fig. S6A. (**E**) An RIP assay was performed in (i) wild-type (WT) E2F1 HCT116 cells treated with 1 μM T1-44 or DMSO for 48 h as indicated, (ii) or in WT E2F1 and E2F1 Cr HCT116 cells. Anti-HNRNPC (HNC), -SRSF2 (SR2), -HNRNPH1 (HNH), or control IgG was used to immunoprecipitate the indicated splicing factors and bound RNA. Recruitment of splicing factors to the indicated exon regions (exon 3, 4, and 5) of *MDM1* are shown. Displayed is the mean percentage enrichment of input, with SD. Significance was calculated by Student’s *t*-test between the indicated sample pairs. (iii) A diagram of the exon (boxed in grey) and intron (black lines) structure around the MDM1 skipped exon 4 (boxed in yellow) is displayed. Specific exon–intron flanking primer pairs used in the RIP analysis are indicated by blue arrows. (iv) A representative immunoblot is included to display input protein levels of E2F1, HNRNPC, SRSF2, HNRNPH1, and SDMe. α-Tubulin was used as a loading control (*n* = 3 biological repeats).

A mechanism that might enable the pRb–E2F pathway to influence alternative splicing is through an interaction with the splicing machinery. To assess this possibility, we performed an investigation using mass spectrometry into the E2F1 interactome. We immunopurified E2F1 from WT HCT116 cells using an anti-E2F1 monoclonal antibody, in the presence or absence of PRMT5 inhibition, recapitulating the treatment conditions where there was an impact on alternative splicing (Fig. 1). As a negative control, we also performed a similar immunopurification in the E2F1 Cr cell line (Fig. 6A). We then searched for proteins in the WT E2F1 interactome that were enriched relative to the negative control cells.

We verified that the immunoprecipitation conditions were specific through the identification of proteins that are known interactors with E2F1, namely DP1, DP2, and pRb (Fig. 6B and C, and Supplementary Table S6). A detailed inspection revealed proteins connected with alternative splicing and RNA processing, including HNRNPC, HNRNPF, PCBP2, and SNRPE, which were evident in the presence or absence of PRMT5 inhibition (Fig. 6B and C, and Supplementary Table S6). SRSF2, a splicing factor previously identified as an E2F1 interacting protein [54], was also observed to be enriched in the E2F1 interactome (Fig. 6B and Supplementary Table S6) though fell just below the threshold for statistical significance in this experiment. We then assessed whether these interactions could be detected by immunoprecipitation, and were able to confirm both SRSF2 and HNRNPC in a complex with endogenous E2F1 (Fig. 6D and Supplementary Fig. S6A).

Since our results suggested that SRSF2 and HNRNPC could form a complex with E2F1, we next examined their recruitment to RNA around the AS exons by RIP. We designed primers across exon–intron boundaries for the skipped exon of *MDM1* (exon 4), and the flanking exons (exon 3 and exon 5), so as to measure recruitment of SRSF2 and HNRNPC to the unspliced, pre-mRNA precursor. RIP signal was compared between WT HCT116 cells treated with DMSO or T1-44 (Fig. 6Ei), or between E2F1 Cr HCT116 cells and the WT controls (Fig. 6Eii). SRSF2 was recruited primarily to the flanking exons, with minimal signal present for exon 4 of *MDM1*. There was also a significant decrease in the recruitment of SRSF2 under conditions of E2F1 loss or PRMT5 inhibition (Fig. 6E). A similar observation was made for HNRNPC after E2F1 loss or PRMT5 inhibition, though enrichment of this splicing factor also occurred at exon 4, albeit at lower levels than that observed for the flanking exons (Fig. 6E). HNRNPH1, selected as a control RNA splicing protein that to our knowledge does not interact with E2F1, was not enriched at any of the *MDM1* exons examined (Fig. 6E). These results indicate that E2F1 and PRMT5 can influence splicing of E2F1 target genes, such as *MDM1*, through the recruitment of splicing factors to pre-mRNA.

To investigate the potential genome wide association between SRSF2/HNRNPC and E2F1- and PRMT5-regulated splicing events, we probed publicly available SRSF2 and HNRNPC CLIP-seq datasets deposited in GEO. SRSF2-binding sites were identified from an SRSF2 PAR-CLIP experiment performed in HeLa and K562 cells [55], whilst HNRNPC-binding sites were identified from an HNRNPC HITS-CLIP experiment performed in HeLa cells [56] or a FLASH-CLIP experiment performed in HEK293 [57]. Binding sites for the two splicing factors were then cross-referenced with the genomic coordinates for significant differential splicing events identified in the rMATS analysis performed on WT E2F1 or E2F1 Cr cells treated with T1-44 (as seen in Fig. 1A–D). The analysis indicated that there were several hundred binding sites for both SRSF2 and HNRNPC that overlapped with E2F1- and PRMT5-regulated AS splice events (Supplementary Fig. S6B and C, and Supplementary Table S7). The proportion of AS events from each treatment condition that intersected with SRSF2-binding sites ranged from 8.4%–9.3% using the HeLa cell dataset, to 13.1%–19.1% using the K562 data (Supplementary Fig. S6B and Supplementary Table S7), whilst 3.2%–4.2% of AS events intersected with HNRNPC-binding sites identified from HeLa cells, or 9.0%–12.0% from HEK293 cells (Supplementary Fig. S6C and Supplementary Table S7). This represented a significant enrichment over the background overlap that would be expected due to random chance, as tested by permutation analysis on randomly shuffled CLIP-seq datasets that maintained the same number, chromosome distribution, and exon/intron distribution of binding sites as the original datasets (Supplementary Fig. S6B and C), suggesting a biologically meaningful association between SRSF2 and HNRNPC with E2F1- and PRMT5-regulated AS events.

**Figure 7.**
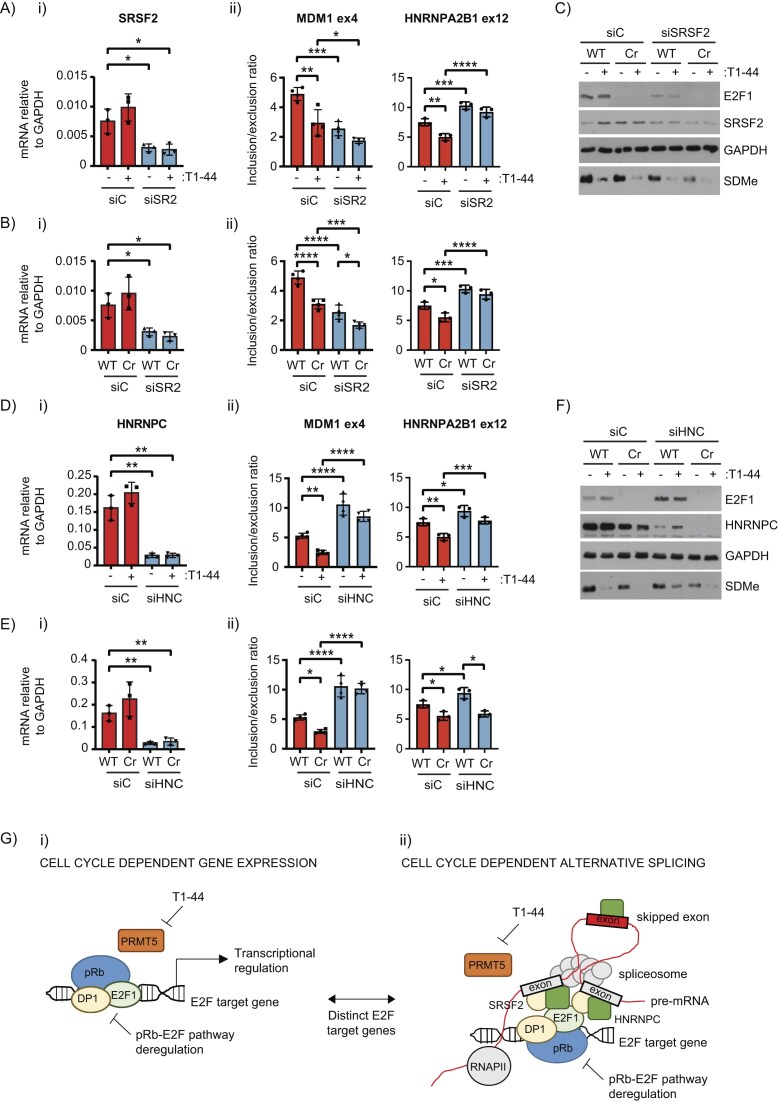
RNA splicing factors contribute to E2F-pathway dependent alternative splicing. (**A**) WT E2F1 HCT116 cells were transfected with control siRNA (siC) or siRNA against SRSF2 (siSR2) prior to treatment with 1 μM T1-44 or DMSO where indicated. (i) RNA extracted from these cells was used in an RT-PCR experiment to monitor the expression of *SRSF2*. Displayed is the mean mRNA expression relative to the *GAPDH* internal calibrator, with SD. Significance was calculated by ANOVA using Sidak’s multiple comparisons test (*n* = 3). (ii) Alternatively, RNA was used to measure the inclusion of *MDM1* exon 4 and *HNRNPA2B1* exon 12 in transcripts. Displayed is the mean inclusion/exclusion ratio with SD. Significance was calculated by ANOVA using Sidak’s multiple comparisons test (biological repeats: *n* = 4 for *MDM1* and *n* = 3 for *HNRNPA2B1*). (**B**) WT E2F1 and E2F1 Cr HCT116 cells were transfected with control siRNA (siC) or siRNA against SRSF2 (siSR2) where indicated. (i) RNA extracted from these cells was used in an RT-PCR experiment to monitor the expression of *SRSF2*. Displayed is the mean mRNA expression relative to the *GAPDH* internal calibrator, with SD. Significance was calculated by ANOVA using Sidak’s multiple comparisons test (*n* = 3). (ii) Alternatively, RNA was used to measure the inclusion of *MDM1* exon 4 and *HNRNPA2B1* exon 12 in transcripts. Displayed is the mean inclusion/exclusion ratio with SD. Significance was calculated by ANOVA using Sidak’s multiple comparisons test (biological repeats: *n* = 4 for *MDM1* and *n* = 3 for *HNRNPA2B1*). (**C**) A representative immunoblot displaying input protein levels of E2F1 and SRSF2 for the experiments described in Fig. 7A and B. SDMe levels are also displayed and GAPDH served as a loading control. (**D**) WT E2F1 HCT116 cells were transfected with control siRNA (siC) or siRNA against HNRNPC (siHNC) prior to treatment with 1 μM T1-44 or DMSO where indicated. (i) RNA extracted from these cells was used in an RT-PCR experiment to monitor the expression of *HNRNPC*. Displayed is the mean mRNA expression relative to the *GAPDH* internal calibrator, with SD. Significance was calculated by ANOVA using Sidak’s multiple comparisons test (*n* = 3). (ii) Alternatively, RNA was used to measure the inclusion of *MDM1* exon 4 and *HNRNPA2B1* exon 12 in transcripts. Displayed is the mean inclusion/exclusion ratio with SD. Significance was calculated by ANOVA using Sidak’s multiple comparisons test (biological repeats: *n* = 4 for *MDM1* and *n* = 3 for *HNRNPA2B1*). (**E**) WT E2F1 and E2F1 Cr HCT116 cells were transfected with control siRNA (siC) or siRNA against HNRNPC (siHNC) where indicated. (i) RNA extracted from these cells was used in an RT-PCR experiment to monitor the expression of *HNRNPC*. Displayed is the mean mRNA expression relative to the *GAPDH* internal calibrator, with SD. Significance was calculated by ANOVA using Sidak’s multiple comparisons test (*n* = 3). (ii) Alternatively, RNA was used to measure the inclusion of *MDM1* exon 4 and *HNRNPA2B1* exon 12 in transcripts. Displayed is the mean inclusion/exclusion ratio with SD. Significance was calculated by ANOVA using Sidak’s multiple comparisons test (biological repeats *n* = 4 for *MDM1* and *n* = 3 for *HNRNPA2B1*). (**F**) A representative immunoblot displaying input protein levels of E2F1 and HNRNPC for the experiments described in Fig. 7D and E. SDMe levels are also displayed and GAPDH served as a loading control. (**G**) Model diagram indicating that the pRb–E2F complex, in concert with PRMT5 activity, regulates cell cycle dependent expression of E2F target genes both at the level of transcriptional control (i), and at the level of AS (ii). E2F target gene networks regulated by transcription or AS tend to be mutually exclusive. AS regulation is achieved in part through interactions between the pRb–E2F complex with RNA splicing factors, including SRSF2 and HNRNPC. E2F and PRMT5 activity regulate the recruitment of these splicing factors to mRNA around AS exons (ii).

We then assessed whether either SRSF2 or HNRNPC impacted on the splicing events influenced by the pRb-E2F pathway. We used siRNA mediated depletion of each protein and measured alternative splicing of candidate genes selected from the overlap between CLIP-seq and rMATS analyses (Fig. 7A–F and Supplementary Fig. S7A–E). We found that the absence of SRSF2 had a significant effect on the splicing, causing reduced or increased exon inclusion in a gene dependent fashion. For example, SRSF2 siRNA treatment reduced the inclusion of *MDM1* exon 4 and *U2AF1* exon 4, but increased the inclusion of *HNRNPA2B1* exon 12, *VCAN* exon 7, *EHMT2* exon 4, and *RTN4* exon 3 (Fig. 7A and B, and Supplementary Fig. S7A and B). For some of the splicing events examined, PRMT5, E2F1, and SRSF2 activity appeared to be mechanistically connected. For example, the reduced inclusion of *MDM1* exon 4 and *HNRNPA2B1* exon 12 observed after T1-44 treatment or E2F1 loss in control siRNA-treated cells was quantitatively reduced or abrogated in cells co-treated with SRSF2 siRNA (Fig. 7A and B). T1-44 treatment regulated *U2AF1* exon 4 and *EHMT2* exon 4 inclusion, an effect which was mimicked by SRSF2 siRNA treatment (Supplementary Fig. S7A). Co-treatment with T1-44 and SRSF2 siRNA had either a quantitatively reduced (*U2AF1* exon 4) or no further effect (*EHMT2* exon 4) on the splice event (Supplementary Fig. S7A). Similarly, loss of E2F1 expression influenced *RTN4* exon 3 inclusion only in control siRNA treated cells. This effect was lost in cells co-treated with SRSF2 siRNA (Supplementary Fig. S7B).

We performed a similar experiment to assess the role of HNRNPC (Fig. 7D and E, and Supplementary Fig. S7C and D). Under conditions of reduced HNRNPC, increased *MDM1* exon 4, *HNRNPA2B1* exon 12, *VCAN* exon 7, *EHMT2* exon 4, and *CREBZF* exon 4 inclusion occurred (Fig. 7D and E, and Supplementary Fig. S7C and D), indicating that HNRNPC was responsible for splicing out of these exons. Conversely, HNRNPC siRNA treatment caused a reduced inclusion of *U2AF1* exon 4 (Supplementary Fig. S7C and D). Treatment with T1-44 reduced the level of exon inclusion in *MDM1* and *HNRNPA2B1*, resembling the effect seen in the E2F1 Cr cells (Fig. 7D and E), though T1-44 did not cause a significant reduction to the inclusion of either exon under HNRNPC siRNA treatment (Fig. 7D). Similarly, T1-44 treatment regulated the splicing of *U2AF1* exon 4 and *EHMT2* exon 4 only in control siRNA treated cells. This effect was lost in cells co-treated with T1-44 and HNRNPC siRNA (Supplementary Fig. S7C). Likewise, E2F1 loss was observed to influence *MDM1* exon 4 and *CREBZF* exon 4 inclusion, though this regulation was quantitatively reduced or lost in cells with reduced HNRNPC expression (Fig. 7E and Supplementary Fig. S7D). Taken together, the results observed in SRSF2 and HNRNPC siRNA-treated cells suggest that, at least for a distinct set of AS events, PRMT5, E2F1, and SRSF2/HNRNPC can act through a common pathway to regulate splicing.

We also investigated whether *SRSF2* and *HNRNPC* expression were dependent on E2F1 in cells, by examining RNA levels derived from each gene in WT and E2F1 Cr cells treated with T1-44 or synchronized by double thymidine block (Supplementary Fig. S7F and G). We did not observe any significant change in *SRSF2* or *HNRNPC* expression upon loss of E2F1, or as cells progressed through the cell cycle from G1 into G2/M phase (Supplementary Fig. S7F and G). Similarly, PRMT5 inhibition had no significant impact on the expression of *HNRNPC*, and caused only a modest insignificant increase in *SRSF2* expression (Supplementary Fig. S7F). These results indicate that changes in the expression of *SRSF2* and *HNRNPC* are unlikely to contribute to the observed regulation of AS by E2F1 and PRMT5 in HCT116 cells. Rather, it is likely to reflect recruitment via E2F1 to the vicinity of the spliced exon.

## Discussion

RNA splicing is a fundamental mechanism by which cells process pre-mRNA to form mature mRNA transcripts that are subsequently translated. Although E2Fs have historically been described as transcription factors which control cell cycle-dependent gene expression, recent work has defined a wider role in both transcriptional control and alternative RNA splicing [24, 28]. Indeed, residue specific arginine methylation by PRMT5 enables E2F1 to regulate RNA splicing of a diverse group of target genes [28]. In part, this is mediated by the tudor domain protein p100/TSN, which reads the methyl mark and allows components of the spliceosome to assemble with E2F1 [28, 29]. Notably, arginine methylation by PRMT5 is also a frequent modification on splicing machinery components; PRMT5-mediated methylation of Sm proteins is important for efficient small nuclear ribonucleoprotein particle (snRNP) assembly [58, 59], whilst targeting of splicing factors like SRSF1 results in an alteration to the subset of mRNAs and splicing associated proteins that it can interact with [60, 61].

With this background in mind, we sought to understand the molecular and biological contribution of E2F pathway components, including pRb and PRMT5, to alternative splicing. This question is of great interest because the pRb–E2F pathway is deregulated in the vast majority of human tumours [6, 8, 10], and *PRMT5* is found to be over-expressed in a broad range of cancers [22, 23]. Deregulation of AS is also a key hallmark of many human diseases, including cancer [30, 32, 33]. Since E2F1 is a specific target for PRMT5 in cells, we reasoned that the interaction between PRMT5 and the broader E2F pathway would provide important information on how abnormal AS activity is achieved in tumours.

A key finding of this study, which arose from multiple genome-wide analyses in CRISPR cell lines lacking E2F pathway components, has established that disruption of the other core components (including pRb and E2F1) results in widespread changes to gene expression, not only at the level of transcription, but also at the level of AS. This we believe documents for the first time that regulation of RNA splicing is a general and widespread effect of pRb–E2F pathway control. Interestingly, genes whose transcription was altered in response to disruption of pRb–E2F pathway components, and genes whose AS changed, were generally represented as distinct mostly non-overlapping gene sets. This is in agreement with previous studies, where using a different approach led to the conclusion that E2F1 has both transcriptional and splicing target genes [28].

Most importantly, we also noticed that the relative contributions of E2F pathway components to AS and transcription were different. Thus, manipulating E2F1 levels demonstrated a greater number of genes regulated at the AS level (as compared to the number of genes regulated at the transcription level), whilst the opposite observation was made when pRb levels were altered where the majority of genes were regulated at the transcription level. However, on a cautionary note, whilst we consider our results to have biological significance, it remains possible that experimental design and the cell tools used in our experiments could influence the output.

Further, a genome-wide analysis performed in wild-type and E2F1 CRISPR cells synchronized at different cell cycle stages identified a remarkably large number of genes (over 4000) with cell cycle-dependent AS changes dependent upon E2F1. Genes regulated by AS during the cell cycle were enriched in biological functions related to cell cycle transition, DNA repair, and cell cycle control. Significantly, the genes under AS control were distinct from the E2F target genes that were under E2F1 transcriptional control. This is in agreement with a previous study, in which mRNA splicing was investigated in synchronously dividing HeLa cells [62].

We also identified splicing events *in situ* in mouse tumours. Namely, we could observe splicing events of specific exons that corresponded to homologues of human E2F1 target genes. These splicing events were also impacted by PRMT5, since tumour bearing mice treated with T1-44 displayed differential splicing of the selected exons. PRMT5 inhibition caused a significant delay to tumour growth in this study [24], suggesting that the differential splicing observed in treated mice might contribute to the decreased tumour growth.

To examine the mechanisms that might connect E2F1 with AS control, we investigated the E2F1 interactome which identified two potential splicing factors as E2F1 interactors. We confirmed that SRSF2 and HNRNPC exist in a complex with endogenous E2F1 and further established their importance in splicing of exons that were dependent on E2F1, pRb, and PRMT5. These same splicing factors were physically recruited to the AS exons influenced by E2F1 and PRMT5.

E2F1 is known to have direct and indirect effects on RNA binding proteins. For example, an interaction between E2F1 and SRSF2 has been observed in lung tumour cell lines, where it is important in regulating entry into S phase and the expression of E2F1 target genes such as *cyclin E* [54]. In addition, *SRSF2* has been identified as an E2F1 target gene, whose expression is regulated in a cell cycle-dependent fashion, particularly in response to DNA damage [63]. In lung adenocarcinoma cell lines, E2F1 required SRSF2 to switch the AS profile of several apoptotic genes towards the expression of pro-apoptotic splice variants [63]. These previous studies are consistent with our results and suggest that the E2F pathway regulates AS in part through direct interaction with the splicing machinery.

In conclusion, our study has established the general and widespread role that the pRb–E2F pathway takes on in transcriptional control and alternative splicing and emphasizes the different groups of genes under each level of control. E2F1-directed AS is regulated throughout the cell cycle, and in response to DNA damage stress, namely two biological contexts widely reported to involve the pRb–E2F pathway. Moreover, E2F1 recruits SRSF2 and HNRNPC to regions of RNA that are targets for AS (Fig. 7G). The pRb–E2F pathway thus impacts on gene expression at multiple interconnected levels.

## Supplementary Material

gkag016_Supplemental_Files

## Data Availability

Lead contact: Requests for further information and resources should be directed to and will be fulfilled by the lead contact, Nicholas La Thangue (nick.lathangue@oncology.ox.ac.uk) Materials availability: All cell lines generated in this study are available from the lead contact without restriction. Data and code availability: The RNA-seq data generated in this manuscript have been deposited in the Gene Expression Omnibus (GEO) under accession code GSE278461. The mass spectrometry data have been deposited in ProteomeXchange consortium via the PRIDE partner repository [64] with the dataset identifier PXD056505. Both datasets are publicly available as of the date of publication. This paper also analyses existing, publicly available RNA-seq data, accessible in GEO under accession codes GSE142430 and GSE181401, and CLIP-seq data accessible in GEO under accession codes GSE207643, GSE138726, and GSE94781. These data do not report original code.
