# Supplementary material for: Separate transcription and splicing gene networks are linked and coordinated by the pRb–E2F pathway
Source: Nucleic Acids Res. 2026 Jan 30;54(3):gkag016. doi: 10.1093/nar/gkag016 (PMC12856215; doi:10.1093/nar/gkag016)
Supplement: gkag016_Supplemental_Files [file gkag016_supplemental_files.zip › SI Materials and Figures - NAR02166C2025R1.pdf]

Supplemental information

Figures S1-S7

Table S1: Excel file containing list of primer sequences used for ChIP, RIP, and QPCR analysis

Table S2: Excel file containing differential gene expression and splicing analysis of HCT116 WT and E2F1 Cr cells treated with T1-44

Table S3: Excel file containing differential gene expression and splicing analysis of MCF7 WT and pRb Cr cells treated with T1-44

Table S4: Excel file containing differential gene expression and splicing analysis of Colon26 derived tumours in mice treated with T1-44

Table S5: Excel file containing differential gene expression and splicing analysis of HCT116 WT and E2F1 Cr cells synchronised by double thymidine block and release

Table S6: Excel file containing E2F1 interactome mass spectrometry analysis

Table S7: Excel file containing overlap analysis between SRSF2/HNRNPC binding sites and alternative splicing events

Figure S1

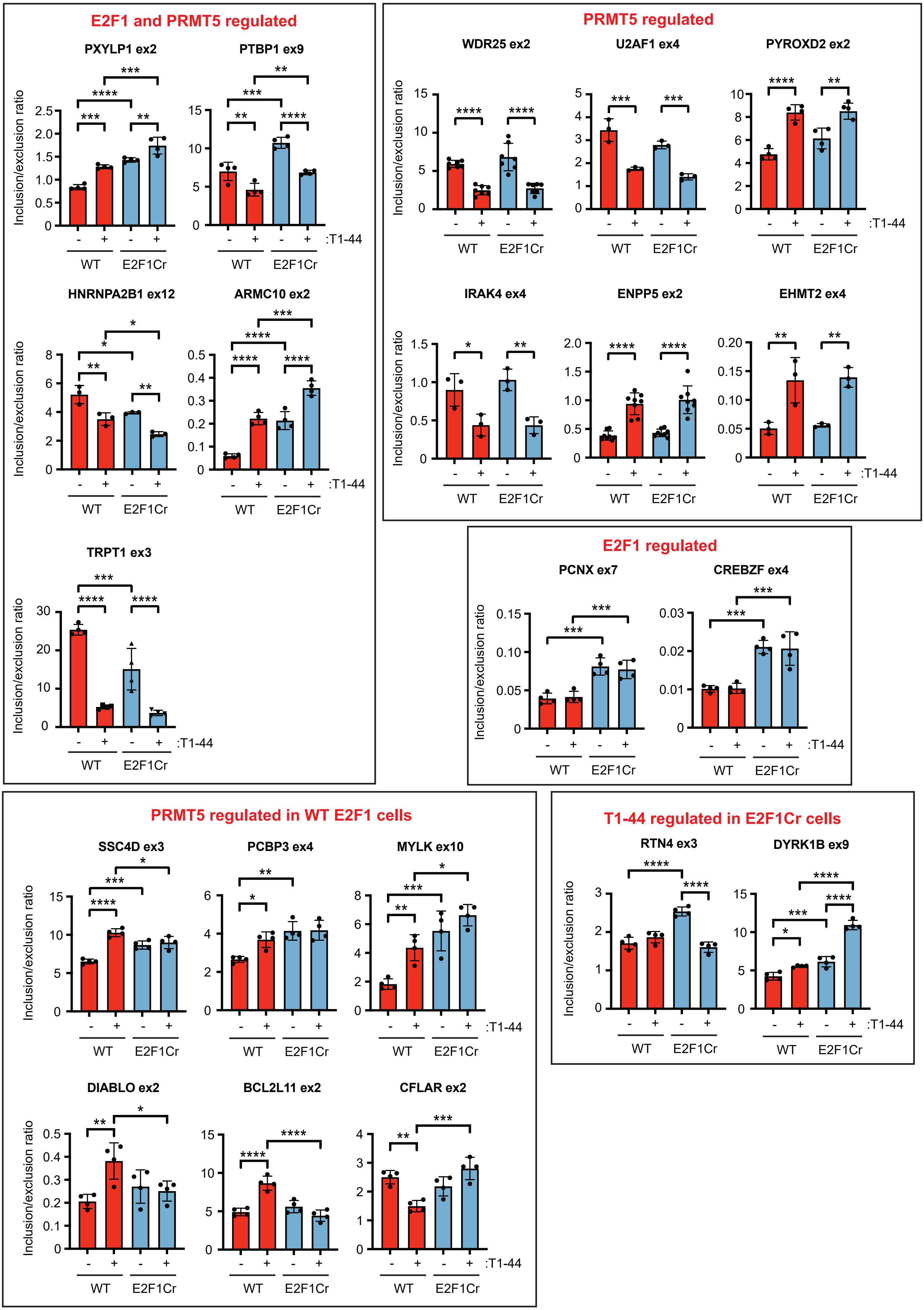

E2F1 and PRMT5 regulated
PXYLP1 ex2
PTBP1 ex9
HNRNPA2B1 ex12
ARMC10 ex2
TRPT1 ex3
PRMT5 regulated
WDR25 ex2
U2AF1 ex4
PYROXD2 ex2
IRAK4 ex4
ENPP5 ex2
EHMT2 ex4
E2F1 regulated
PCNX ex7
CREBZF ex4
PRMT5 regulated in WT E2F1 cells
SSC4D ex3
PCBP3 ex4
MYLK ex10
DIABLO ex2
BCL2L11 ex2
CFLAR ex2
T1-44 regulated in E2F1Cr cells
RTN4 ex3
DYRK1B ex9
Inclusion/exclusion ratio
- + - + :T1-44
WT
E2F1Cr

Figure S1: RT-PCR analysis, related to Figure 1

Wild-type (WT) E2F1 and E2F1 Cr HCT116 cells were treated for 48 h with 1 μM T1-44 or DMSO as indicated. An RT-PCR was performed to measure the inclusion of the indicated exons in RNA transcripts from the cells. Displayed is the mean inclusion/exclusion ratio, with SD. The data has been categorised into groupings determined by whether the splice events is regulated by PRMT5 alone, E2F1 alone, by both E2F1 and PRMT5, or by PRMT5 only in a WT E2F1 or E2F1 Cr background. Significance was calculated by ANOVA using Sidak's multiple comparisons test. These results are from the same experiment represented in Figure 1K. (n = 4 biological repeats, except for *HNRNPA2B1*, *U2AF1*, *IRAK4* and *EHMT2* where n = 3, and *WDR25* and *ENPP5* where n = 7)

FIGURE S2

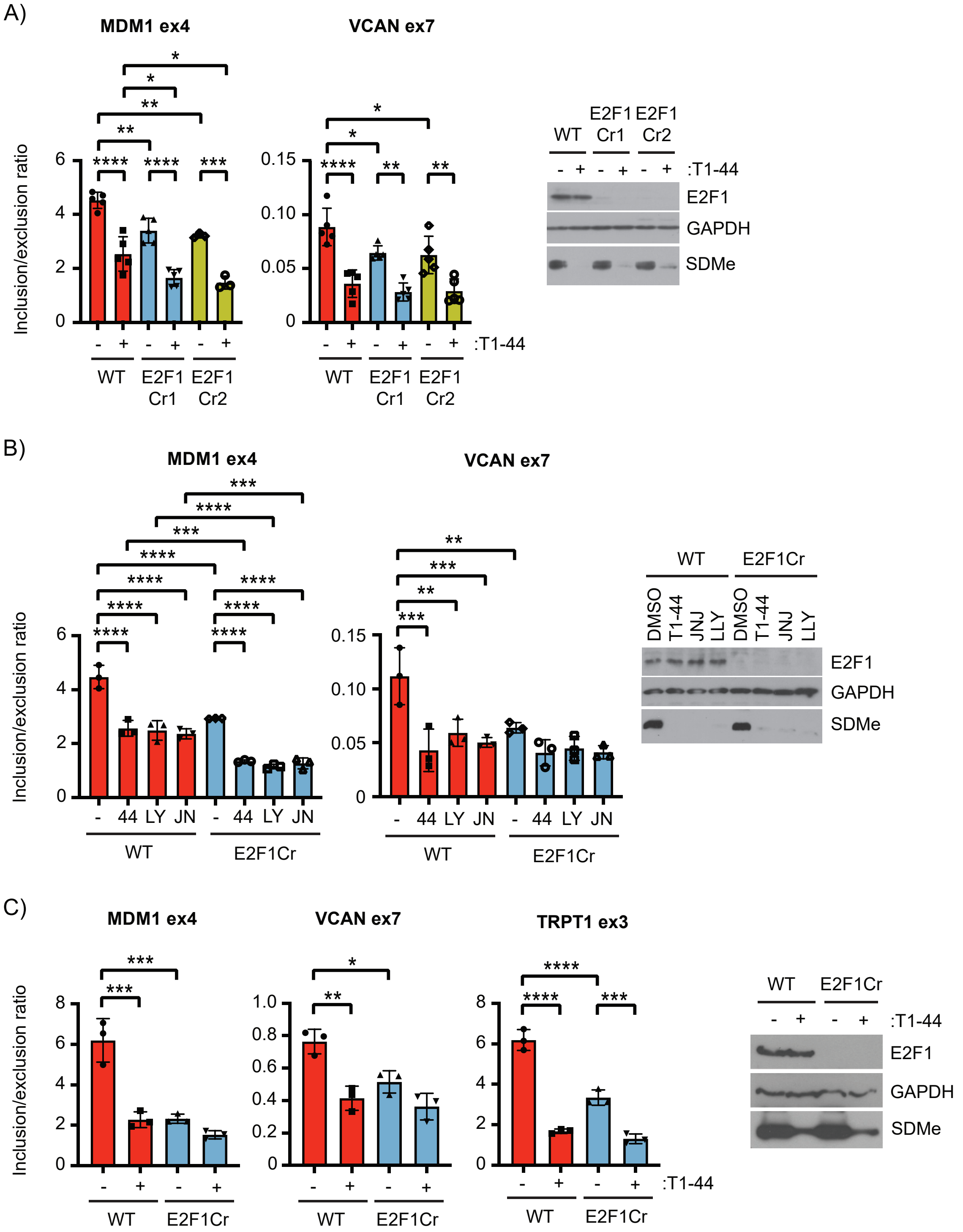

A)
MDM1 ex4
VCAN ex7
Inclusion/exclusion ratio
:T1-44
WT
E2F1 Cr1
E2F1 Cr2
E2F1
GAPDH
SDMe
B)
MDM1 ex4
VCAN ex7
- 44 LY JN
WT
E2F1Cr
DMSO
T1-44
JNJ
LLY
C)
MDM1 ex4
VCAN ex7
TRPT1 ex3

Figure S2: RT-PCR analysis, related to Figure 1

A. Wild-type (WT) E2F1 and two independent E2F1 Cr HCT116 cell clones (Cr1 and Cr2) were treated for 48 h with 1 μM T1-44 or DMSO as indicated. An RT-PCR was performed to measure the inclusion of *MDM1* exon 4 or *VCAN* exon 7 in RNA transcripts from the cells. Displayed is the mean inclusion/exclusion ratio, with SD. Significance was calculated by ANOVA using Sidak's multiple comparisons test. A representative immunoblot is included to display input protein levels of E2F1 and symmetric dimethylation (SDMe). GAPDH was used as a loading control. (n = 5 biological repeats)

B. Wild-type (WT) E2F1 and E2F1 Cr HCT116 cells were treated for 48 h with 1 μM T1-44, LLY-283, JNJ-64619178 or DMSO as indicated. An RT-PCR was performed to measure the inclusion of *MDM1* exon 4 or *VCAN* exon 7 in RNA transcripts from the cells. Displayed is the mean inclusion/exclusion ratio, with SD. Significance was calculated by ANOVA using Sidak's multiple comparisons test. A representative immunoblot is included to display input protein levels of E2F1 and symmetric dimethylation (SDMe). GAPDH was used as a loading control. (n = 3 biological repeats)

C. Wild-type (WT) E2F1 and E2F1 Cr PANC1 cells were treated for 48 h with 1 μM T1-44 or DMSO as indicated. An RT-PCR was performed to measure the inclusion of *MDM1* exon 4, *VCAN* exon 7, or *TRPT1* exon 3 in RNA transcripts from the cells. Displayed is the mean inclusion/exclusion ratio, with SD. Significance was calculated by ANOVA using Sidak's multiple comparisons test. A representative immunoblot is included to display input protein levels of E2F1 and symmetric dimethylation (SDMe). GAPDH was used as a loading control. (n = 3 biological repeats)

FIGURE S3

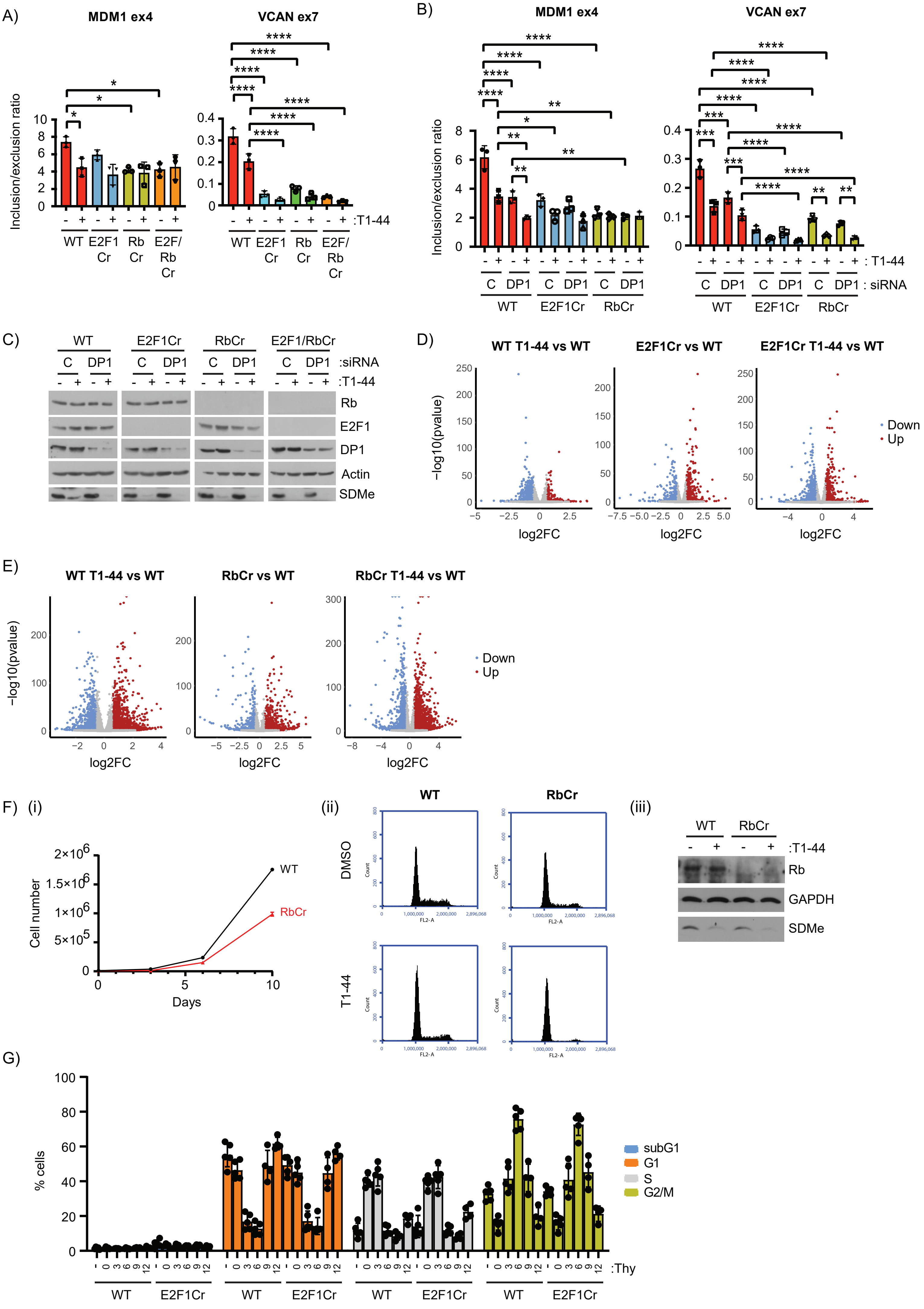
A)
MDM1 ex4
VCAN ex7
Inclusion/exclusion ratio
:T1-44
WT
E2F1 Cr
Rb Cr
E2F/ Rb Cr
B)
MDM1 ex4
VCAN ex7
Inclusion/exclusion ratio
: T1-44
C
DP1
: siRNA
WT
E2F1Cr
RbCr
C)
WT
E2F1Cr
RbCr
E2F1/RbCr
:siRNA
:T1-44
Rb
E2F1
DP1
Actin
SDMe
D)
WT T1-44 vs WT
E2F1Cr vs WT
E2F1Cr T1-44 vs WT
−log10(pvalue)
log2FC
Down
Up
E)
WT T1-44 vs WT
RbCr vs WT
RbCr T1-44 vs WT
−log10(pvalue)
log2FC
Down
Up
F) (i)
Cell number
Days
WT
RbCr
(ii)
WT
RbCr
DMSO
T1-44
FL2-A
Count
(iii)
WT
RbCr
:T1-44
Rb
GAPDH
SDMe
G)
% cells
:Thy
WT
E2F1Cr
subG1
G1
S
G2/M

Figure S3: RT-PCR and gene expression analysis, related to Figures 1 and 2

A. Wild-type (WT) or E2F1 Cr, Rb Cr, and E2F1/Rb Cr HCT116 cells were treated for 48 h with 1 μM T1-44 or DMSO as indicated. An RT-PCR was performed to measure the inclusion of *MDM1* exon 4 or *VCAN* exon 7 in RNA transcripts from the cells. Displayed is the mean inclusion/exclusion ratio, with SD. Significance was calculated by ANOVA using Sidak's multiple comparisons test. (n = 3 biological repeats)

B. Wild-type (WT) or E2F1 Cr and Rb Cr HCT116 cells were transfected with siRNA targeting *DP1* for 72 h where indicated. Cells were also treated for 48 h with 1 μM T1-44 or DMSO. An RT-PCR was performed to measure the inclusion of *MDM1* exon 4 or *VCAN* exon 7 in RNA transcripts from the cells. Displayed is the mean inclusion/exclusion ratio, with SD. Significance was calculated by ANOVA using Sidak's multiple comparisons test. (n = 3 biological repeats)

C. A representative immunoblot displaying input protein levels of Rb, E2F1, DP1 and symmetric demethylation (SDMe) in wild-type (WT), E2F1 Cr, Rb Cr, and E2F1/Rb Cr HCT116 cells transfected with siRNA targeting *DP1* for 72 h where indicated. Cells were also treated for 48 h with 1 μM T1-44 or DMSO. Actin was used as a loading control. This immunoblot accompanies the experiments performed in Figure S3A and S3B.

D. Volcano plots displaying values of log2 fold change and –log10 p values for differentially expressed genes identified between the indicated treatments and wild-type (WT) E2F1 HCT116 cells. Red colour represents genes upregulated in the treated cells, whilst blue colour represents genes downregulated in treated cells. Grey colour represents genes that fell below the fold change or statistical cut-off applied (padj < 0.05, log2FC > 0.58). These data were derived from the same RNA-seq analysis used to generate Figures 2A and 2B.

E. Volcano plots displaying values of log2 fold change and –log10 p values for differentially expressed genes identified between the indicated treatments and wild-type (WT) pRb MCF7 cells. Red colour represents genes upregulated in the treated cells, whilst blue colour represents genes downregulated in treated cells. Grey colour represents genes that fell below the fold change or statistical cut-off applied

(padj < 0.05, log2FC > 0.58). These data were derived from the same RNA-seq analysis used to generate Figures 2D and 2E.

F. (i) A representative growth curve for wild-type (WT) Rb MCF7 and Rb Cr cells. Cells were seeded at an initial density of 1 x 10^4 cells and followed for 10 days. Displayed are mean cell numbers from technical duplicates with SD. (n = 3 biological repeats) (ii) Wild-type (WT) Rb MCF7 and Rb Cr cells were treated with 1 μM T1-44 for 48 h and analysed for cell cycle profile analysis by flow cytometry of propidium iodide stained cells. Displayed is a representative FACS profile for each treatment. (n = 3 biological repeats) (iii) A representative immunoblot is also included to demonstrate input Rb and symmetric dimethylation (SDMe) levels. GAPDH was used as a loading control.

G. WT E2F1 and E2F1 Cr HCT116 cells were synchronised at the G1/S boundary with a double thymidine block. Cells were released from the block for the indicated number of hours and a cell cycle profile analysis was performed by flow cytometry of propidium iodide stained cells. Displayed is the mean percentage of cells observed in G1, S, G2/M and sub G1 phases, with SD shown. This experiment accompanies the analysis performed in Figure 4A. (n = 4 biological repeats)

FIGURE S4

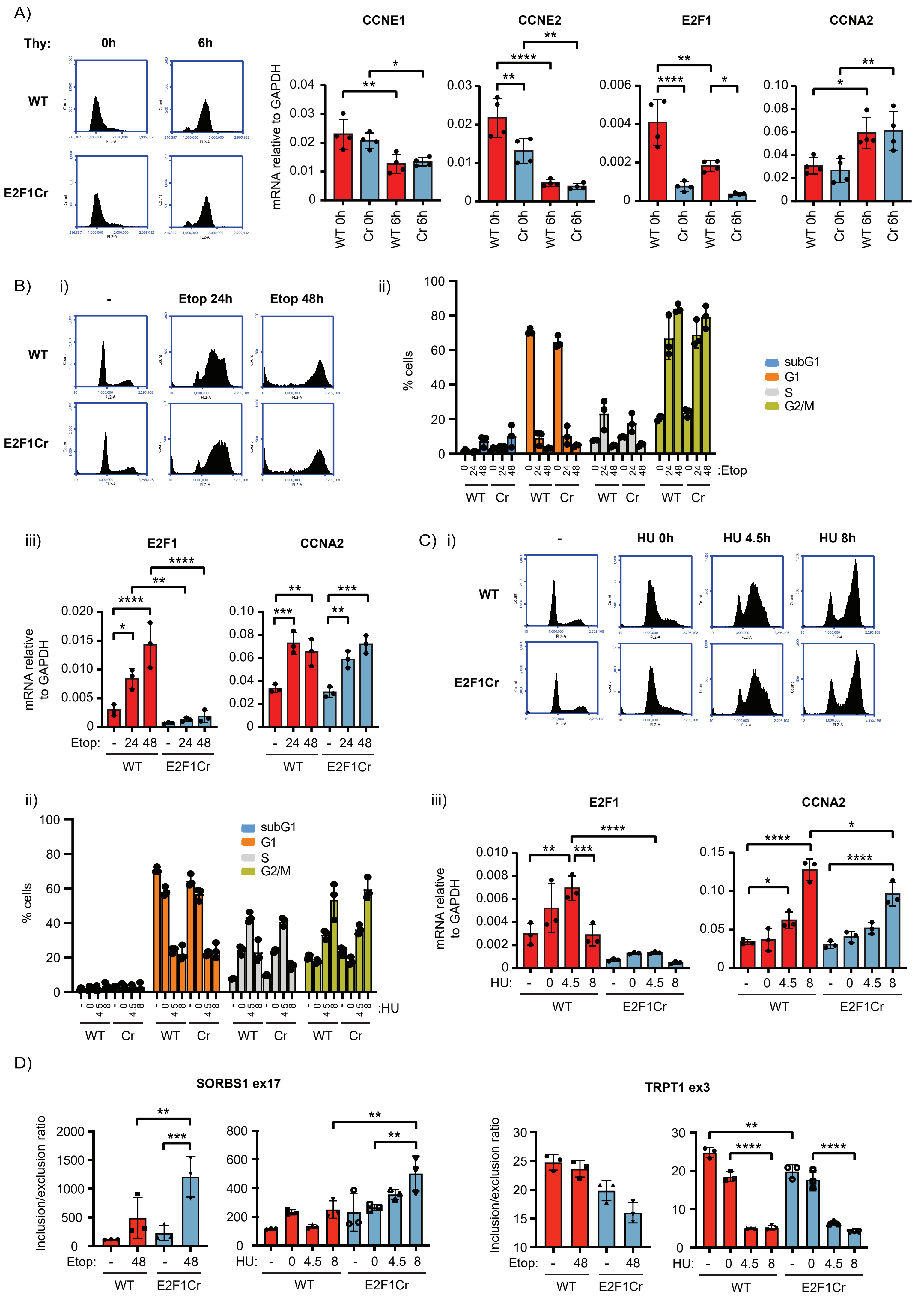
A)
Thy:
0h
6h
WT
E2F1Cr
CCNE1
CCNE2
E2F1
CCNA2
mRNA relative to GAPDH
WT 0h
Cr 0h
WT 6h
Cr 6h
B)
i)
-
Etop 24h
Etop 48h
ii)
% cells
subG1
G1
S
G2/M
:Etop
iii)
Etop:
C)
HU 0h
HU 4.5h
HU 8h
:HU
HU:
D)
SORBS1 ex17
TRPT1 ex3
Inclusion/exclusion ratio

Figure S4: Cell cycle and RT-PCR analysis, related to Figures 4 and 5

A. On the left, a representative flow cytometry profile for wild-type (WT) E2F1 and E2F1 Cr HCT116 cells synchronised at the G1/S boundary with a double thymidine block (0 h), or released from the block to progress into G2/M phase (6 h), are displayed. On the right, RNA extracted from these treated cells was used in an RT-PCR experiment to measure the expression of E2F1 target genes (*CCNE1*, *CCNE2*, *E2F1, CCNA2*) known to be regulated in a cell cycle dependent fashion. Displayed is the mean mRNA expression relative to the *GAPDH* internal calibrator, with SD. Significance was calculated by ANOVA using Sidak's multiple comparisons test. These samples are the same as those used in Figure 4, and support the analysis performed in Figure 4A and Figure 5. (n = 4 biological repeats)

B. (i) Representative flow cytometry profiles for wild-type (WT) E2F1 and E2F1 Cr HCT116 cells treated with 20 μM etoposide for 24 or 48h where indicated. (ii) Cell cycle analysis was performed by flow cytometry of propidium iodide stained cells. Displayed is the mean percentage of cells observed in G1, S, G2/M and sub G1 phases, with SD shown. (iii) Alternatively, RNA was extracted from these treated cells and used in an RT-PCR experiment to measure the expression of *E2F1* and *CCNA2*. Displayed is the mean mRNA expression relative to the *GAPDH* internal calibrator, with SD. These samples are the same as those used in Figure 4B. (n = 3 biological repeats)

C. (i) Representative flow cytometry profiles for wild-type (WT) E2F1 and E2F1 Cr HCT116 cells treated with 1 mM etoposide for 24h to synchronise cells at the G1/S boundary (HU 0h). Cells were then released from the block for the indicated number of hours to allow cells to progress through S phase (HU 4.5h) into G2/M (HU 8h). (ii) Cell cycle analysis was performed by flow cytometry of propidium iodide stained cells. Displayed is the mean percentage of cells observed in G1, S, G2/M and sub G1 phases, with SD shown. (ii) Alternatively, RNA was extracted from these treated cells and used in an RT-PCR experiment to measure the expression of *E2F1* and *CCNA2*. Displayed is the mean mRNA expression relative to the *GAPDH* internal calibrator, with SD. These samples are the same as those used in Figure 4B. (n = 3 biological repeats)

D. Wild-type (WT) E2F1 and E2F1 Cr HCT116 cells were treated with 20 μM etoposide for 48 h, or with 1 mM hydroxyurea (HU) for 24 h, where indicated. HU treated cells were then released from the block for the indicated number of hours. RNA was extracted and an RT-PCR was performed to measure the inclusion of *SORBS1* exon 17 or *TRPT1* exon 3 in RNA transcripts. Displayed is the mean inclusion/exclusion ratio, with SD. Significance was calculated by ANOVA using Sidak's multiple comparisons test. (n = 3 biological repeats). This data accompanies the analysis performed in Figure 4B.

FIGURE S5

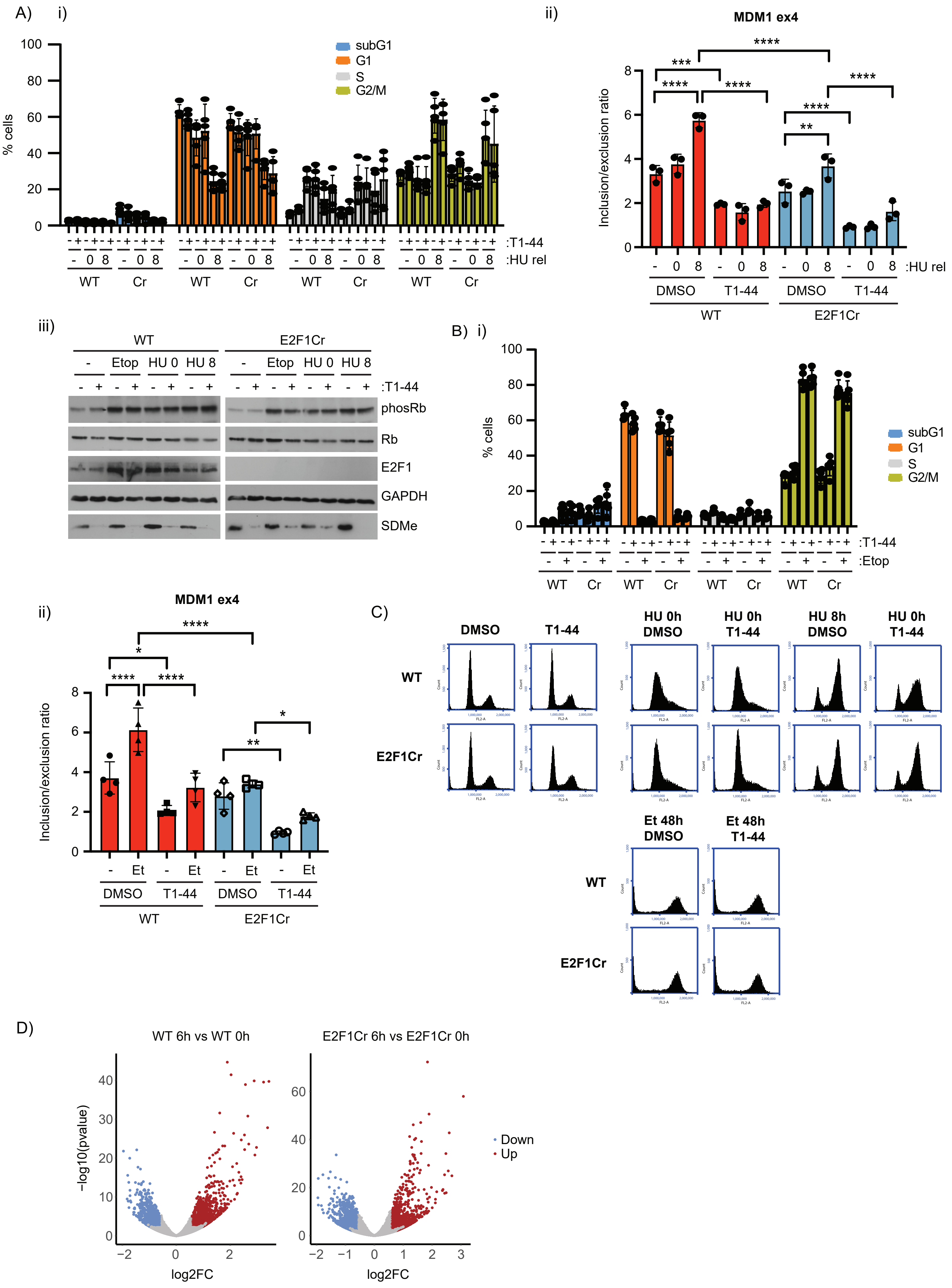

A) i)
% cells
subG1
G1
S
G2/M
:T1-44
:HU rel
WT
Cr
ii)
MDM1 ex4
Inclusion/exclusion ratio
:HU rel
DMSO
T1-44
WT
E2F1Cr
iii)
WT
E2F1Cr
Etop
HU 0
HU 8
:T1-44
phosRb
Rb
E2F1
GAPDH
SDMe
B) i)
% cells
subG1
G1
S
G2/M
:T1-44
:Etop
ii)
MDM1 ex4
Inclusion/exclusion ratio
Et
DMSO
T1-44
WT
E2F1Cr
C)
DMSO
T1-44
HU 0h DMSO
HU 0h T1-44
HU 8h DMSO
HU 0h T1-44
WT
E2F1Cr
Et 48h DMSO
Et 48h T1-44
D)
WT 6h vs WT 0h
E2F1Cr 6h vs E2F1Cr 0h
−log10(pvalue)
log2FC
Down
Up

Figure S5: RT-PCR data analysis, related to Figure 4

A. (i) Wild-type (WT) E2F1 and E2F1 Cr HCT116 cells were treated with 1 μM T1-44 or DMSO for 48 h as indicated. They were subsequently treated with 1 mM hydroxyurea (HU) for 24 h to synchronise cells at the G1/S boundary. Cells were then released from the block for the indicated number of hours and a cell cycle analysis was performed by flow cytometry of propidium iodide stained cells. Displayed is the mean percentage of cells observed in G1, S, G2/M and sub G1 phases, with SD shown. (ii) Alternatively, RNA was extracted from these treated cells and used in an RT-PCR experiment to measure the inclusion of *MDM1* exon 4 in RNA transcripts from the cells. Displayed is the mean inclusion/exclusion ratio, with SD. Significance was calculated by ANOVA using Sidak's multiple comparisons test. (iii) A representative immunoblot is included to display input protein levels of E2F1, Rb, phosphorylated Rb (phosRb) and symmetric dimethylation (SDMe). GAPDH was used as a loading control. This immunoblot also contains samples treated for 48 h with 20 μM etoposide (Etop) that are part of Figure S5B. (n = 4 biological repeats)

B. (i) Wild-type (WT) E2F1 and E2F1 Cr HCT116 cells were treated with 1 μM T1-44 or DMSO for 48 h as indicated, along with a co-treatment for 48 h with 20 μM etoposide (Etop) where displayed. A cell cycle analysis was performed by flow cytometry of propidium iodide stained cells. Displayed is the mean percentage of cells observed in G1, S, G2/M and sub G1 phases, with SD shown. (ii) Alternatively, RNA was extracted from these treated cells and used in an RT-PCR experiment to measure the inclusion of *MDM1* exon 4 in RNA transcripts from the cells. Displayed is the mean inclusion/exclusion ratio, with individual data points. Significance was calculated by ANOVA using Sidak's multiple comparisons test. An immunoblot displaying input protein levels of E2F1, Rb, phosphorylated Rb (phosRb) and symmetric dimethylation (SDMe) is displayed in Figure S5A (iii). (n = 4 biological repeats)

C. Representative flow cytometry profiles for wild-type (WT) E2F1 and E2F1 Cr HCT116 cells treated with DMSO or T1-44, and etoposide (Et) or hydroxyurea (HU) as described for Figure S5A and S5B.

D. Volcano plots displaying values of log2 fold change and –log10 p values for differentially expressed genes identified between the indicated treatments of wild-type (WT) E2F1 and E2F1 Cr HCT116 cells synchronised in G1/S (0 h) or G2/M (6 h) by double thymidine block and release. Red colour represents genes upregulated in the treated cells, whilst blue colour represents genes downregulated in treated cells. Grey colour represents genes that fell below the fold change or statistical cut-off applied (padj < 0.05, log2FC > 0.58). These data were derived from the same RNA-seq analysis used to generate Figure 4H. (n = 4 biological repeats)

FIGURE S6

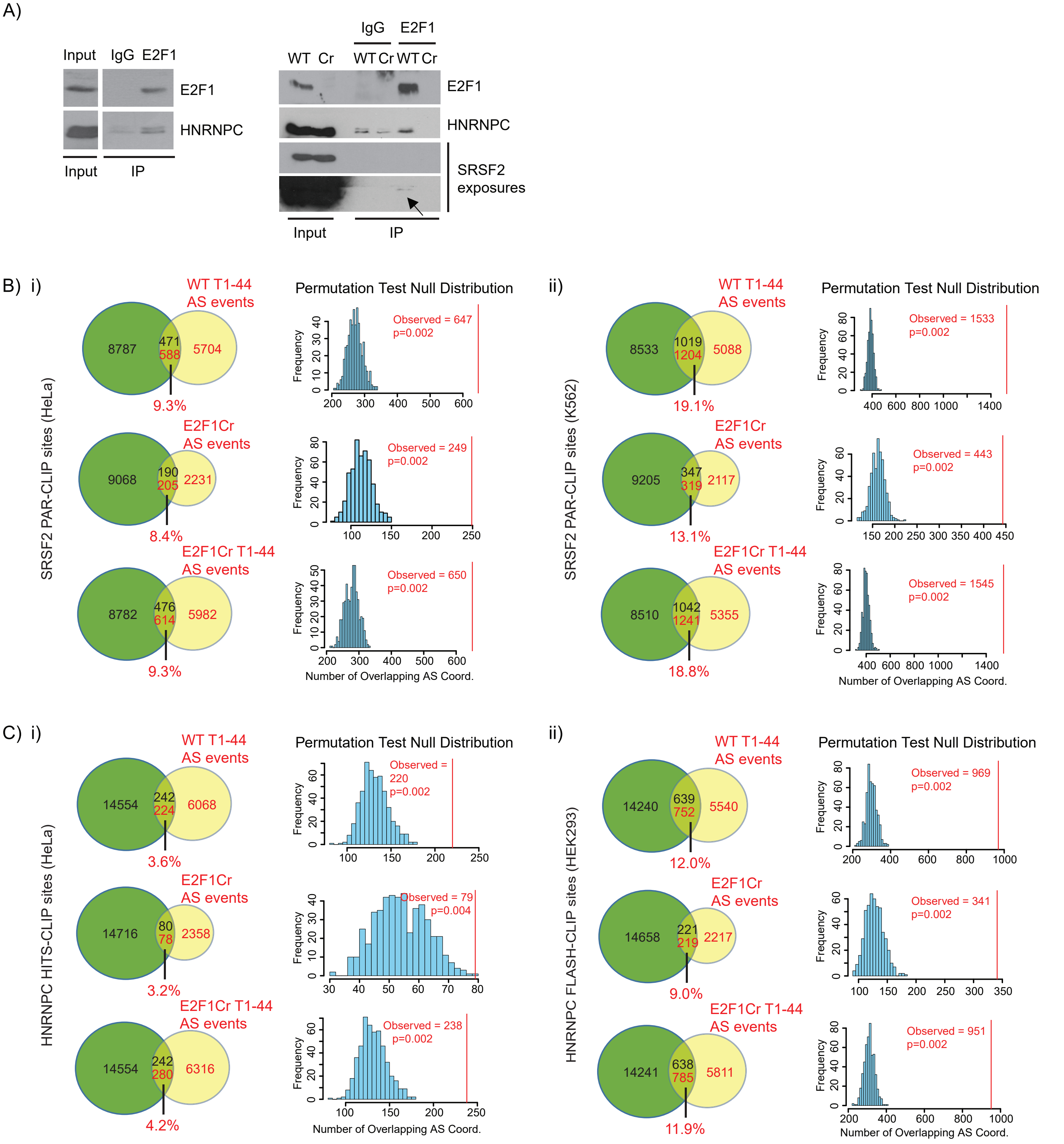
A)
Input
IgG
E2F1
E2F1
HNRNPC
Input
IP
IgG
E2F1
WT
Cr
WT
Cr
WT
Cr
E2F1
HNRNPC
SRSF2
exposures
Input
IP
B) i)
SRSF2 PAR-CLIP sites (HeLa)
WT T1-44
AS events
8787
471
588
5704
9.3%
Permutation Test Null Distribution
Observed = 647
p=0.002
Frequency
E2F1Cr
AS events
9068
190
205
2231
8.4%
Observed = 249
p=0.002
E2F1Cr T1-44
AS events
8782
476
614
5982
9.3%
Observed = 650
p=0.002
Number of Overlapping AS Coord.
ii)
SRSF2 PAR-CLIP sites (K562)
WT T1-44
AS events
8533
1019
1204
5088
19.1%
Permutation Test Null Distribution
Observed = 1533
p=0.002
E2F1Cr
AS events
9205
347
319
2117
13.1%
Observed = 443
p=0.002
E2F1Cr T1-44
AS events
8510
1042
1241
5355
18.8%
Observed = 1545
p=0.002
Number of Overlapping AS Coord.
C) i)
HNRNPC HITS-CLIP sites (HeLa)
WT T1-44
AS events
14554
242
224
6068
3.6%
Permutation Test Null Distribution
Observed =
220
p=0.002
E2F1Cr
AS events
14716
80
78
2358
3.2%
Observed = 79
p=0.004
E2F1Cr T1-44
AS events
14554
242
280
6316
4.2%
Observed = 238
p=0.002
Number of Overlapping AS Coord.
ii)
HNRNPC FLASH-CLIP sites (HEK293)
WT T1-44
AS events
14240
639
752
5540
12.0%
Permutation Test Null Distribution
Observed = 969
p=0.002
E2F1Cr
AS events
14658
221
219
2217
9.0%
Observed = 341
p=0.002
E2F1Cr T1-44
AS events
14241
638
785
5811
11.9%
Observed = 951
p=0.002
Number of Overlapping AS Coord.

Figure S6: Immunoprecipitation and CLIP binding site/AS associated region overlap analyses, related to Figure 6

A. An immunoprecipitation experiment (shown on the left) was performed in wild-type E2F1 HCT116 cells using an anti-E2F1 antibody or control IgG as indicated. Interacting HNRNPC was detected using specific antibodies. Input protein levels are also displayed. An independent immunoprecipitation experiment (shown on the right) was performed in wild-type (WT) E2F1 or E2F1 Cr HCT116 cells using an anti-E2F1 antibody or control IgG as indicated. Interacting HNRNPC and SRSF2 was detected using specific antibodies (two different exposures for SRSF2 are displayed). Input protein levels are also shown. These are additional representative examples of E2F1 immunoprecipitations to accompany the experiment performed in Figure 6D.

B. AS coordinates for significant differential splicing events output for each treatment from the HCT116 RNA-seq rMATS analysis (Figure 1A), were overlapped with SRSF2 CLIP sites identified in HeLa (i) or K562 cells (ii) (GSE207643), allowing for ±200bp to account for the potential influence of nearby splicing factor binding on exon/intron splicing. Venn diagrams display the overlap of SRSF2 binding coordinates (numbers in black text), with specific AS events in each treatment (numbers in red text). The percentage below each venn represents the proportion of AS events in each treatment that have overlapping SRSF2 binding sites. To the right of each venn is a graph displaying the null distribution of overlapping AS coordinates determined from 500 permutations of shuffled SRSF2 CLIP sites. The vertical red line represents the observed number of overlapping AS coordinates with the original SRSF2 CLIP dataset, with the Monte-Carlo p-value displayed to assess enrichment significance.

C. AS coordinates for significant differential splicing events output for each treatment from the HCT116 RNA-seq rMATS analysis (Figure 1A), were overlapped with HNRNPC CLIP sites identified in HeLa (i) (GSE138726) or HEK293 cells (ii) (GSE94781), allowing for ±200bp to account for the potential influence of nearby splicing factor binding on exon/intron splicing. Venn diagrams display the overlap of HNRNPC binding coordinates (numbers in black text), with specific AS events in each treatment (numbers in red text). The percentage below each venn represents the proportion of AS events in each treatment that have overlapping HNRNPC

binding sites. To the right of each venn is a graph displaying the null distribution of overlapping AS coordinates determined from 500 permutations of shuffled HNRNPC CLIP sites. The vertical red line represents the observed number of overlapping AS coordinates with the original HNRNPC CLIP dataset, with the Monte-Carlo p-value displayed to assess enrichment significance.

FIGURE S7

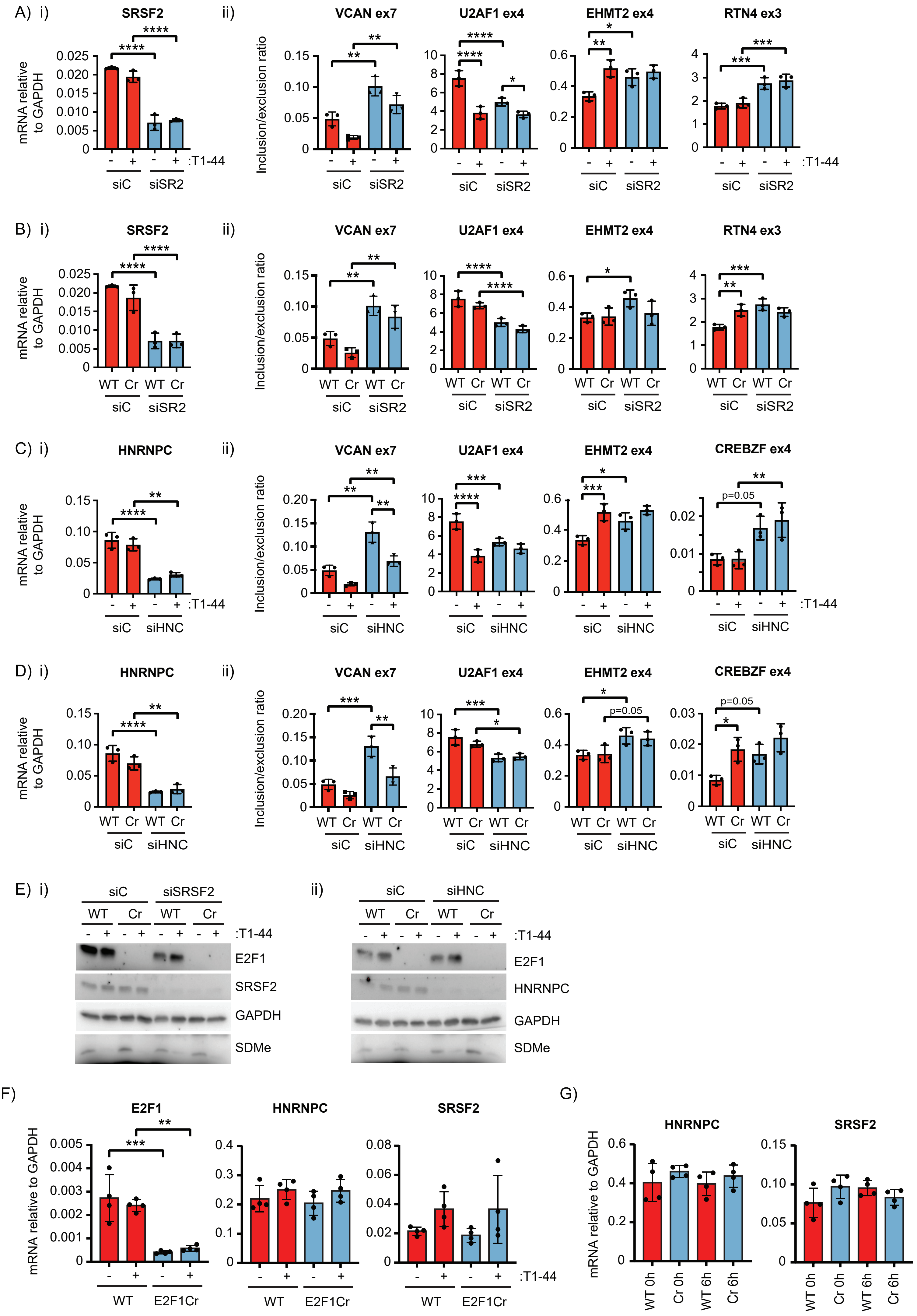

A) i) SRSF2
mRNA relative to GAPDH
:T1-44
siC siSR2
ii) VCAN ex7 U2AF1 ex4 EHMT2 ex4 RTN4 ex3
Inclusion/exclusion ratio
B) i) SRSF2
WT Cr WT Cr
ii) VCAN ex7 U2AF1 ex4 EHMT2 ex4 RTN4 ex3
C) i) HNRNPC
siC siHNC
ii) VCAN ex7 U2AF1 ex4 EHMT2 ex4 CREBZF ex4
p=0.05
D) i) HNRNPC
ii) VCAN ex7 U2AF1 ex4 EHMT2 ex4 CREBZF ex4
p=0.05
E) i) siC siSRSF2
WT Cr WT Cr
:T1-44
E2F1
SRSF2
GAPDH
SDMe
ii) siC siHNC
E2F1
HNRNPC
GAPDH
SDMe
F) E2F1 HNRNPC SRSF2
mRNA relative to GAPDH
WT E2F1Cr
:T1-44
G) HNRNPC SRSF2
mRNA relative to GAPDH
WT 0h Cr 0h WT 6h Cr 6h

Figure S7: RT-PCR analysis, related to Figure 7

A. WT E2F1 HCT116 cells were transfected with control siRNA (siC) or siRNA against SRSF2 (siSR2) prior to treatment with 1 μM T1-44 or DMSO where indicated. (i) RNA extracted from these cells was used in an RT-PCR experiment to monitor the expression of *SRSF2.* Displayed is the mean mRNA expression relative to the *GAPDH* internal calibrator, with SD. Significance was calculated by ANOVA using Sidak's multiple comparisons test. (n = 3) (ii) Alternatively, RNA was used to measure the inclusion of *VCAN* exon 7, *U2AF1* exon 4, *EHMT2* exon 4 and *RTN4* exon 3 in transcripts. Displayed is the mean inclusion/exclusion ratio with SD. Significance was calculated by ANOVA using Sidak's multiple comparisons test. (biological repeats: n = 3). This data accompanies the analysis in Figure 7A.

B. WT E2F1 and E2F1 Cr HCT116 cells were transfected with control siRNA (siC) or siRNA against SRSF2 (siSR2) where indicated. (i) RNA extracted from these cells was used in an RT-PCR experiment to monitor the expression of *SRSF2.* Displayed is the mean mRNA expression relative to the *GAPDH* internal calibrator, with SD. Significance was calculated by ANOVA using Sidak's multiple comparisons test. (n = 3) (ii) Alternatively, RNA was used to measure the inclusion of *VCAN* exon 7, *U2AF1* exon 4, *EHMT2* exon 4 and *RTN4* exon 3 in transcripts. Displayed is the mean inclusion/exclusion ratio with SD. Significance was calculated by ANOVA using Sidak's multiple comparisons test. (biological repeats: n = 3). This data accompanies the analysis in Figure 7B.

C. WT E2F1 HCT116 cells were transfected with control siRNA (siC) or siRNA against HNRNPC (siHNC) prior to treatment with 1 μM T1-44 or DMSO where indicated. (i) RNA extracted from these cells was used in an RT-PCR experiment to monitor the expression of *HNRNPC.* Displayed is the mean mRNA expression relative to the *GAPDH* internal calibrator, with SD. Significance was calculated by ANOVA using Sidak's multiple comparisons test. (n = 3) (ii) Alternatively, RNA was used to measure the inclusion of *VCAN* exon 7, *U2AF1* exon 4, *EHMT2* exon 4 and *CREBZF* exon 4 in transcripts. Displayed is the mean inclusion/exclusion ratio with SD. Significance was calculated by ANOVA using Sidak's multiple comparisons test. (biological repeats: n = 3). This data accompanies the analysis in Figure 7D.

D. WT E2F1 and E2F1 Cr HCT116 cells were transfected with control siRNA (siC) or siRNA against HNRNPC (siHNC) where indicated. (i) RNA extracted from these cells was used in an RT-PCR experiment to monitor the expression of *HNRNPC.* Displayed is the mean mRNA expression relative to the *GAPDH* internal calibrator, with SD. Significance was calculated by ANOVA using Sidak's multiple comparisons test. (n = 3) (ii) Alternatively, RNA was used to measure the inclusion of *VCAN* exon 7, *U2AF1* exon 4, *EHMT2* exon 4 and *CREBZF* exon 4 in transcripts. Displayed is the mean inclusion/exclusion ratio with SD. Significance was calculated by ANOVA using Sidak's multiple comparisons test. (biological repeats: n = 3). This data accompanies the analysis in Figure 7E.

E. Representative immunoblots displaying input protein levels of E2F1 and SRSF2 (i) or HNRNPC (ii) for the experiments described in SI Figure 7A to 7D. Symmetric dimethylation (SDMe) levels are also displayed and GAPDH served as a loading control.

F. RNA from wild-type (WT) E2F1 and E2F1 Cr cells treated with 1 μM T1-44 or DMSO for 48 h as indicated was used in an RT-PCR experiment to monitor the expression of *E2F1*, *HNRNPC*, and *SRSF2*. Displayed is the mean mRNA expression relative to the *GAPDH* internal calibrator, with SD. Significance was calculated by ANOVA using Sidak's multiple comparisons test. This RT-PCR experiment uses the same samples as those displayed in Figure 1K. (n = 4 biological repeats)

G. RNA from WT E2F1 and E2F1 Cr cells synchronised either in G1/S (0 h) or G2/M (6 h) using a double thymidine block were used in an RT-PCR experiment to monitor the expression of *HNRNPC* and *SRSF2*. Displayed is the mean mRNA expression relative to the *GAPDH* internal calibrator, with SD. This RT-PCR experiment uses the same samples as those displayed in Figure 5. (n = 4 biological repeats)
